# The F-box E3 ubiquitin ligase BAF1 mediates the degradation of the brassinosteroid-activated transcription factor BES1 through selective autophagy in Arabidopsis

**DOI:** 10.1093/plcell/koab210

**Published:** 2021-08-26

**Authors:** Ping Wang, Trevor M Nolan, Natalie M Clark, Hao Jiang, Christian Montes-Serey, Hongqing Guo, Diane C Bassham, Justin W Walley, Yanhai Yin

**Affiliations:** 1 Department of Genetics, Development and Cell Biology, Iowa State University, Ames, Iowa 50011; 2 Department of Plant Pathology and Microbiology, Iowa State University, Ames, Iowa 50011; 3 Plant Sciences Institutes, Iowa State University, Ames, Iowa 50011

## Abstract

Brassinosteroids (BRs) regulate plant growth, development, and stress responses by activating the core transcription factor BRI1-EMS-SUPPRESSOR1 (BES1), whose degradation occurs through the proteasome and autophagy pathways. The E3 ubiquitin ligase(s) that modify BES1 for autophagy-mediated degradation remain to be fully defined. Here, we identified an F-box family E3 ubiquitin ligase named BES1-ASSOCIATED F-BOX1 (BAF1) in *Arabidopsis thaliana*. BAF1 interacts with BES1 and mediates its ubiquitination and degradation. Our genetic data demonstrated that BAF1 inhibits BR signaling in a BES1-dependent manner. Moreover, BAF1 targets BES1 for autophagic degradation in a selective manner. BAF1-triggered selective autophagy of BES1 depends on the ubiquitin binding receptor DOMINANT SUPPRESSOR OF KAR2 (DSK2). Sucrose starvation-induced selective autophagy of BES1, but not bulk autophagy, was significantly compromised in *baf1* mutant and *BAF1-ΔF* (BAF1 F-box decoy) overexpression plants, but clearly increased by *BAF1* overexpression. The *baf1* and *BAF1-ΔF* overexpression plants had increased BR-regulated growth but were sensitive to long-term sucrose starvation, while *BAF1* overexpression plants had decreased BR-regulated growth but were highly tolerant of sucrose starvation. Our results not only established BAF1 as an E3 ubiquitin ligase that targets BES1 for degradation through selective autophagy pathway, but also revealed a mechanism for plants to reduce growth during sucrose starvation.

## Teaser

During sucrose starvation, BAF1-mediated degradation of the transcription factor BES1 via selective autophagy inhibits brassinosteroid signaling.

##  

**Figure:**
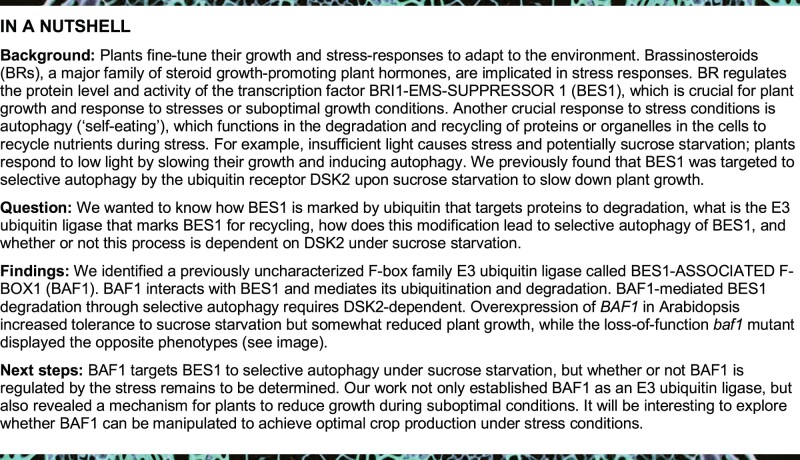


## Introduction

Brassinosteroids (BRs) are a family of polyhydroxylated plant steroid hormones that play crucial roles in many important growth and developmental processes, as well as in stress responses (Clouse et al., 1996; Nolan et al., 2020). BRs are perceived by the cell-surface plasma membrane-localized receptor kinases BR INSENSITIVE1 (BRI1) and its homologs BRI1-LIKE1 (BRL1) and BRI1-LIKE3 (BRL3), along with co-receptor BRI1-ASSOCIATED KINASE1 (BAK1) and its homologs. The binding of BRs to BRI1 and BAK1 ultimately initiates a signaling cascade to activate the downstream transcription factors BRI1-EMS-SUPPRESSOR1 (BES1) and BRASSINAZOLE-RESISTANT1 (BZR1) (Clouse et al., 1996; Li and Chory, 1997; Li and Nam, 2002; Li et al., 2002; Wang et al., 2002; Yin et al., 2002). In the absence of BRs, the negative regulator BRASSINOSTEROID-INSENSITIVE2 (BIN2), a glycogen synthase kinase-3-like (GSK3-like) kinase, phosphorylates BES1 and BZR1, leading to their inactivation through multiple mechanisms (He et al., 2002; Li and Jin, 2007). In the presence of BRs, BR SIGNALING KINASES (BSKs) and CONSTITUTIVE DIFFERENTIAL GROWTH1 (CDG1) are phosphorylated by BRI1 and activate the phosphatase BRI1 SUPPRESSOR1 (BSU1), which was proposed to dephosphorylate and inhibit BIN2 (Tang et al., 2008; Kim et al., 2011). The inactivation of BIN2 and the action of PROTEIN PHOSPHATASE 2A (PP2A) lead to the dephosphorylation of BES1 and BZR1 and their accumulation in the nucleus, where they interact with other transcriptional regulators to control the expression of thousands of BR-regulated genes (He et al., 2002; Yin et al., 2002, 2005; Wang et al., 2002; Tang et al., 2011; Sun et al., 2010; Guo et al., 2013; Yu et al., 2011).

BES1 and BZR1 are not only key transcriptional factors in the BR signaling pathway, but also serve as hubs that integrate diverse signals to regulate plant growth, development and adaptability to environmental changes (Yang and Wang, 2017; Nolan et al., 2020). The *bes1-D* and *bzr1-D* mutants were identified as gain-of-function mutations associated with dramatic accumulation of BES1 and BZR1 proteins and constitutive BR responses (He et al., 2002; Wang et al., 2002; Yin et al., 2002), indicating that BES1 and BZR1 are subject to posttranslational regulation and their protein stability is crucial for their function.

Ubiquitin-mediated control of protein stability is central to most aspects of plant development and homeostasis (Smalle and Vierstra, 2004; Kelley, 2018). The specificity of protein ubiquitination is mainly determined by E3 ubiquitin ligases, as they provide recognition and binding specificity to the substrate in a temporally and spatially regulated manner (Vierstra, 2009). The S PHASE KINASE-ASSOCIATED PROTEIN1 (SKP1)-Cullin-F-Box (SCF) E3 ubiquitin ligases have been extensively studied as key regulators in plant hormone signaling (Vierstra, 2009; Kelley and Estelle, 2012). Several E3 ubiquitin ligases involved in the degradation of BES1 and BZR1 have been identified. The F-box protein MORE AXILLARY GROWTH LOCUS 2 (MAX2) ubiquitinates BES1 in response to strigolactone signaling to suppress shoot branching (Wang et al., 2013). Both phosphorylated and dephosphorylated BES1 can interact with and be degraded by MAX2 (Wang et al., 2013). Two other RING type E3 ubiquitin ligases, CONSTITUTIVE PHOTOMORPHOGENIC 1 (COP1) and SINA of *Arabidopsis thaliana* (SINATs), are involved in dark/light-mediated BES1 and BZR1 degradation (Kim et al., 2014; Yang et al., 2017). COP1 mediates the degradation of the inactive phosphorylated BZR1 in the dark, leading to the accumulation of dephosphorylated BZR1 to promote hypocotyl elongation (Kim et al., 2014), whereas SINATs specifically target dephosphorylated and active BES1 in the light to inhibit hypocotyl elongation (Yang et al., 2017). A plant U-box E3 ubiquitin ligase, PUB40, was recently reported to degrade BZR1 in a root-specific manner and mediates root response to inorganic phosphate deprivation (Kim et al., 2019). These studies indicate that the regulation of BES1 and BZR1 protein stability is tissue-specific and developmental or environmental context-dependent.

Upon ubiquitination, proteins can be targeted for degradation through either proteasome or autophagy pathways (Floyd et al., 2012). In addition to being degraded through the proteasome pathway, BES1 and BZR1 can also be targeted for autophagy-mediated degradation (Zhang et al., 2016; Nolan et al., 2017). Sugar signaling promotes the accumulation of BZR1 through the TARGET OF RAPAMYCIN (TOR) pathway to promote plant growth, while sugar starvation-triggered TOR inactivation leads to autophagy-mediated BZR1 degradation to inhibit plant growth (Zhang et al., 2016). The mechanism underlying autophagy-mediated BES1 degradation was established upon the identification of DOMINANT SUPPRESSOR OF KAR2 (DSK2) (Nolan et al., 2017). DSK2 is a ubiquitin receptor, and it interacts with ubiquitinated BES1 and the autophagy protein ATG8 to target BES1 for autophagy-mediated degradation under drought or sucrose starvation conditions (Nolan et al., 2017). BIN2 phosphorylates DSK2 and promotes DSK2-ATG8 interaction. However, the E3 ubiquitin ligases that target BES1 for autophagy-mediated degradation via ubiquitination remain to be further defined.

In this study, we identified an F-box family E3 ubiquitin ligase involved in autophagy-mediated BES1 protein degradation that we termed BES1-ASSOCIATED F-BOX1 (BAF1). We confirmed that BES1 and BAF1 interact in vitro and in vivo. Furthermore, we found that BAF1 can promote the ubiquitination and degradation of BES1 both in *Nicotiana benthamiana* and in *A.* *thaliana* plants. Endogenous BES1 stability and protein levels were greatly reduced in *BAF1* overexpression plants and increased in *baf1* loss-of-function mutants and *BAF1-ΔF* (BAF1 F-box decoy) overexpression plants, with correspondingly altered responses to brassinolide (BL) and the BR biosynthesis inhibitor brassinozole (BRZ). Biochemical and genetic studies indicated that BAF1 mediates BES1 degradation through selective autophagy in a DSK2-dependent manner. Selective autophagy of BES1 induced by sucrose starvation was significantly compromised in *baf1* and *BAF1-ΔF* overexpression plants but was increased by *BAF1* overexpression. However, BAF1 did not affect bulk autophagy induced by either sucrose starvation or nitrogen starvation. The *baf1* and *BAF1-ΔF* overexpression plants had increased BR-regulated growth but were sensitive to long-term sucrose starvation, while *BAF1* overexpression plants had decreased BR-regulated growth but were highly tolerant of sucrose starvation. Our studies thus establish BAF1 as an E3 ubiquitin ligase for BES1 and provide new insights into the mechanisms through which BES1 protein is degraded by selective autophagy under sucrose starvation.

## Results

### BAF1 interacts with BES1 in vitro and in vivo

**Figure 1:**
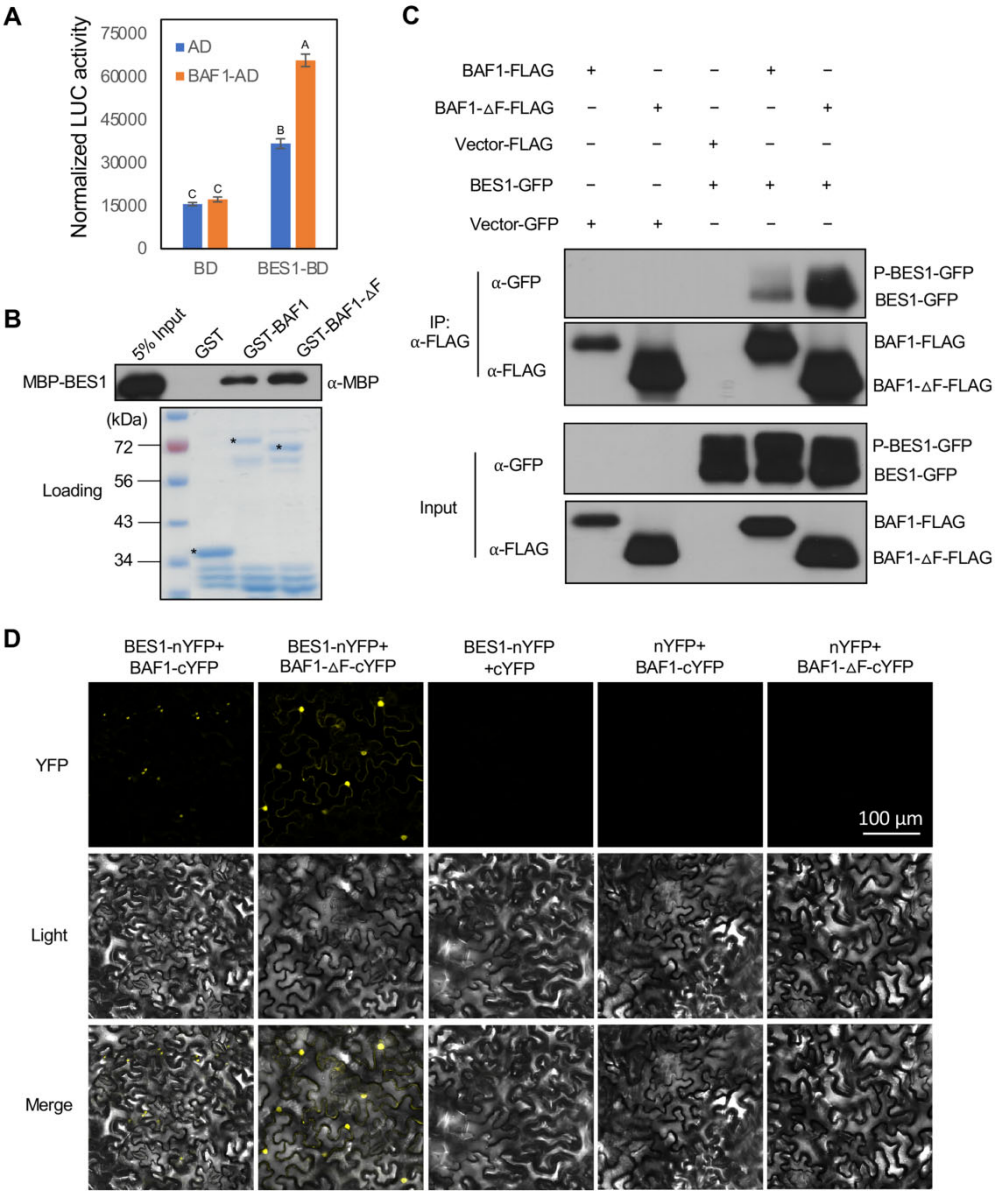
BAF1 interacts with BES1. A, BAF1 interacted with BES1 in yeast as detected by β-galactosidase activity which was assessed using a commercial luminescent β-galactosidase substrate Beta-Glo. The GAL4 DNA BD of pGBKT7 was fused to N-terminus of full-length BES1 protein, and the GAL4 AD of pGADT7 was fused to N-terminus of full-length BAF1 protein. Data represent mean ± SEM, *n* = 8. Different letters indicate significant difference according to one-way ANOVA Tukey’s multiple range tests (*P* < 0.05). B, BAF1 interacted with BES1 in GST pull-down assay. The loading of GST, GST-BAF1, and GST-BAF1-ΔF proteins were shown by a Coomassie-stained gel (bottom). Asterisks indicated the desired proteins. MBP-BES1 was detected by immunoblotting with anti-MBP (mouse) antibody. C, Co-IP assay showed BAF1 and BES1 interaction in Arabidopsis protoplasts. BAF1-FLAG, BAF1-ΔF-FLAG, and BES1-GFP as well as control vectors were co-transformed into Arabidopsis protoplasts. Protein was immunoprecipitated with anti-FLAG (mouse) and detected with anti-FLAG (rabbit) and anti-GFP (rabbit) antibodies. D, BAF1 interacted with BES1 by BiFC in *N. benthamiana*. Fluorescence, light, or merged images of leaf cells are shown. Scale bar represents 100 µm.

From a yeast-two-hybrid (Y2H) screen targeting potential BES1 interactors involved in protein degradation (Yin et al., 2005; Nolan et al., 2017), we identified an F-box family E3 ubiquitin ligase that we termed BES1-Associated F-box 1 (BAF1), encoded by *At1g76920*. We first confirmed this interaction using full-length BES1 and BAF1 in yeast (Figure 1, A). BAF1 protein is 374 amino acids in length, containing an F-box domain at the N terminus and one Kelch domain (Supplemental Figure S1, A). To investigate the role of the F-box domain in BAF1-mediated BES1 degradation, we generated a truncated version of BAF1 that lacks the F-box domain (BAF1-ΔF, Supplemental Figure S1, A). We expected that BAF1-ΔF can still interact with BES1 but would be unable to recruit it to the SCF complex for degradation. This approach has been shown to generate dominant-negative “decoy” for other F-box E3 ligases (Feke et al., 2019).

We tested the interaction of BES1 with BAF1 in vitro using glutathione *S*-transferase (GST) pull-down assays and found that both GST-BAF1 and GST-BAF1-ΔF directly interacted with BES1 (Figure 1, B). To map the regions of BES1 that mediate its interaction with BAF1, different BES1 domains (Supplemental Figure S1, E) fused with maltose-binding protein (MBP) were used for in vitro GST pull-down assays. We found that both GST-BAF1 and GST-BAF1-ΔF had strong interactions with the PEST domain and C-terminal domain of BES1 (Supplemental Figure S1, F). All the purified proteins used for GST pull-down assays are shown in Supplemental Figure S1, G.

To confirm the interaction of BES1 and BAF1 in planta, we performed co-immunoprecipitation (Co-IP) assays in Arabidopsis protoplasts. BAF1-FLAG and BAF1-ΔF-FLAG co-immunoprecipitated both dephosphorylated BES1-GFP and phosphorylated BES1-GFP (P-BES1-GFP), but the vector control did not (Figure 1, C andSupplemental Figure S1, H). The interaction was also confirmed using bimolecular fluorescence complementation (BiFC) assays in *N.* *benthamiana* leaves. While very weak signals from small puncta were observed when full-length BAF1 and BES1 were co-expressed, a strong YFP signal was observed in the nucleus when BAF1-ΔF and BES1 were co-expressed, as well as some signal in the cytoplasm (Figure 1, D). We speculated that BES1 might be degraded when co-expressed with full-length BAF1, while BES1 is stabilized by BAF1-ΔF. Immunoblotting of the BiFC samples confirmed the expression of each construct, and also showed that BES1-nYFP protein level was decreased when co-expressed with full-length BAF1-cYFP but not with BAF1-ΔF-cYFP (Supplemental Figure S1, I). Taken together, these data indicate that BAF1 physically interacts with BES1 both in vitro and in vivo.

### BAF1 mediates the ubiquitination and degradation of BES1

**Figure 2:**
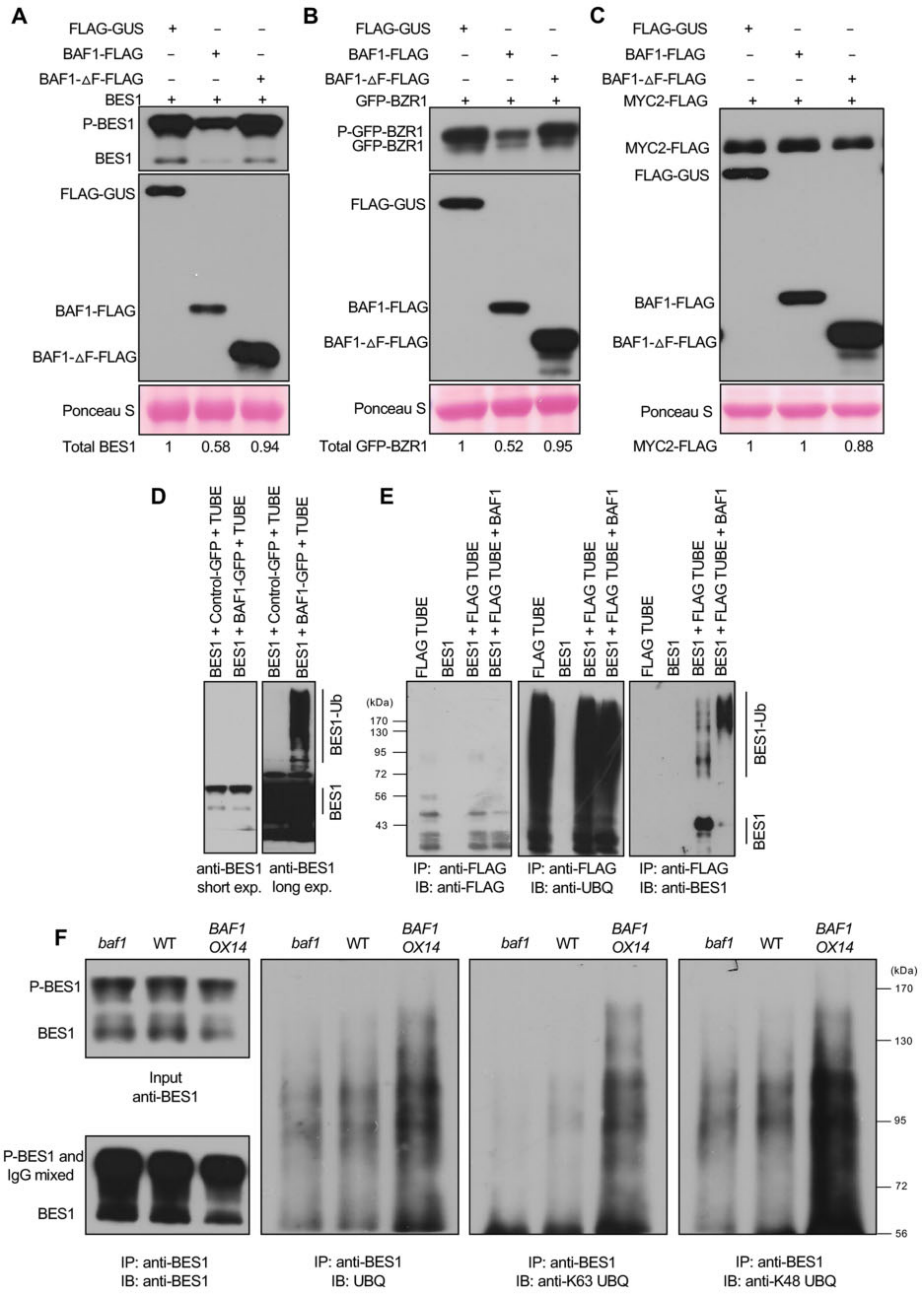
BAF1 mediates BES1 degradation and ubiquitination in *N. benthamiana*. A, BAF1 degraded BES1 while BAF1-ΔF stabilized BES1 in *N. benthamiana*. BAF1-FLAG, BAF1-ΔF-FLAG, and BES1 as well as control vectors were co-infiltrated into *N. benthamiana* for 2 days. Proteins were detected by immunoblotting with anti-BES1 and anti-FLAG (rabbit) antibodies. B, BAF1 also degraded GFP-BZR1 while BAF1-ΔF stabilized it in *N. benthamiana*. BAF1-FLAG, BAF1-ΔF-FLAG, and GFP-BZR1 as well as control vectors were co-infiltrated into *N. benthamiana* for 2 days. Proteins were detected by immunoblotting with anti-GFP (rabbit) and anti-FLAG (rabbit) antibodies. C, BAF1 did not degrade MYC2-FLAG in *N. benthamiana*. BAF1-FLAG, BAF1-ΔF-FLAG, and MYC2-FLAG as well as control vectors were co-infiltrated into *N. benthamiana* for 2 days. Proteins were detected by immunoblotting with anti-FLAG (rabbit) antibody. D, Observation of BES1 ubiquitinated by BAF1 with TUBE. BAF1-GFP, TUBE-FLAG, and BES1 as well as control vectors were co-infiltrated into *N. benthamiana* for 2 days. Proteins were detected by immunoblotting with anti-BES1 antibody for short and long exposure. E, Ubiquitination of BES1 in *N. benthamiana*. BAF1-GFP, TUBE-FLAG, and BES1 as well as control vectors were co-infiltrated into *N. benthamiana* for 2 days. Proteins were immunoprecipitated with anti-FLAG (mouse) and analyzed by immunoblotting with anti-FLAG (rabbit), anti-UBQ (chicken), and anti-BES1 antibodies. F, In vivo K63- and K48-linked ubiquitination of BES1 from WT, *baf1*, and *BAF1 OX14* seedlings. BES1 protein was immunoprecipitated from 7-day-old seedlings using BES1 antibody. The immunoprecipitation product was analyzed by immunoblotting with anti-BES1, anti-K63-UBQ, and anti-K48-UBQ antibodies, respectively. The size of P (phosphorylated)-BES1 is close to that of rabbit IgG protein, so when detecting the immunoprecipitation product by anti-BES1 antibody, P-BES, and IgG co-migrate, but the unphosphorylated BES1 band can be seen clearly, indicating the successful immunoprecipitation. Ponceau S staining of total protein serves as loading control (Rubisco band is shown). Quantified relative band intensity was listed below using Image J.

The aforementioned interactions indicated that BAF1 may function as an E3 ubiquitin ligase for BES1. To determine whether or not BAF1 can mediate BES1 degradation, we co-expressed BES1 with FLAG-tagged BAF1 or BAF1-ΔF in *N. benthamiana* leaves. Consistent with our observations in BiFC assays, BES1 protein levels were decreased by 50% upon co-expression with BAF1 but not with BAF1-ΔF (Figure 2, A). We also performed these assays using BES1-D, which has a proline-to-leucine residue mutation at position 233 in the PEST domain and this mutation stabilizes BES1 protein (Yin et al., 2002). Interestingly, BAF1 could also reduce BES1-D-GFP protein level when co-expressed in *N. benthamiana* (Supplemental Figure S3, A). BAF1 also interacted with BES1-D in yeast (Supplemental Figure S3, B), suggesting that the PEST domain mutation in BES1-D did not disrupt BES1–BAF1 interaction. BES1 shares 88% identity with its homolog BZR1 (Yin et al., 2002). To test if BAF1 also mediates BZR1 degradation, we co-expressed BAF1-FLAG and BZR1-GFP in *N. benthamiana*. BAF1 reduced the BZR1-GFP protein level by half while BAF1-ΔF stabilized it, indicating that BAF1 can likely also target BZR1 (Figure 2, B). However, BAF1 did not interact with and had no effect on the transcription factor MYC2, which is phosphorylated and destabilized by FERONIA receptor kinase (Guo et al., 2018; Supplemental Figure S2 and Figure 2, C). These results indicated that BAF1 functions specifically in BES1 and BZR1 protein degradation.

To test if the E3 ubiquitin ligase BAF1 mediates ubiquitination of BES1, we transiently expressed BES1 and BAF1 in *N. benthamiana* together with tandem ubiquitin binding entities (TUBEs), a tandem array of four ubiquitin-associated domains (UBAs) from human Ubiquilin 1 that binds to polyubiquitinated proteins and protects them from degradation and deubiquitination (Yoshida et al., 2015). We found that in the presence of BAF1, high-molecular weight forms of BES1 extensively accumulated compared with the control, which is consistent with an increase in BES1 ubiquitination by BAF1 (Figure 2, D). To confirm this finding, we immunoprecipitated FLAG-TUBE from the *N. benthamiana* leaves with anti-FLAG, which enriched high-molecular weight forms of BES1 that cross-reacted with anti-BES1and anti-Ubiquitin antibodies (Figure 2, E). Furthermore, when BAF1 was introduced, BES1 showed a greater shift, likely due to increased ubiquitination (Figure 2, E). These results indicate that BAF1 promotes BES1 ubiquitination in *N. benthamiana*.

To characterize the function of BAF1 in Arabidopsis, we identified a T-DNA insertion knock-out mutant *baf1* (Supplemental Figure S1, A and B) and generated *35S:BAF1*-*FLAG* overexpression lines (referred to as *OX14* and *OX16* hereafter) and dominant negative *35S:BAF1-ΔF-FLAG* overexpression lines (referred to as *ΔF OX18* and *ΔF OX46*). Consistent with a role for BAF1 in BES1 protein degradation, *OX14* and *OX16* lines had reduced BES1 protein, while *ΔF OX18* and *ΔF OX46* had more BES1 compared with WT (Supplemental Figure S1, C). It is worth noting that *BES1* transcript was not affected in *baf1* mutant, *BAF1 OX14* or *BAF1-ΔF OX46* lines compared with WT, indicating that BAF1 regulates BES1 through post-translational modification (Supplemental Figure S1, D).

To further confirm BAF1-mediated BES1 ubiquitination in planta, we immunoprecipitated endogenous BES1 from 7-day-old WT, *baf1* and *OX14* seedlings using anti-BES1 antibody. Relatively less ubiquitinated BES1 was detected by anti-UBQ antibody in the *baf1* mutant compared with WT (Supplemental Figure S3, C and Figure 2, F), while there was much more ubiquitinated BES1 in *BAF1* overexpression plants (Supplemental Figure S3, D and Figure 2, F). These results showed that ubiquitination of BES1 is affected by BAF1 in plants.

Proteomic analysis in Arabidopsis revealed that the two most abundant polyubiquitylation linkages are Lysine 48 (K48) and Lysine 63 (K63) (Kim et al., 2013). To investigate whether BAF1-mediated BES1 undergoes K48- or K63-linked ubiquitination, we performed the same BES1 immunoprecipitation assay with anti-K63-UBQ and anti-K48-UBQ antibodies, respectively. BES1 ubiquitination was detected with both anti-K63-UBQ and anti-K48-UBQ antibodies, with a slightly lower level in the *baf1* mutant but much higher levels in *BAF1* overexpression plants compared with WT (Figure 2, F). These results indicated that BES1 undergoes both K63- and K48-linked ubiquitination.

**Figure 3:**
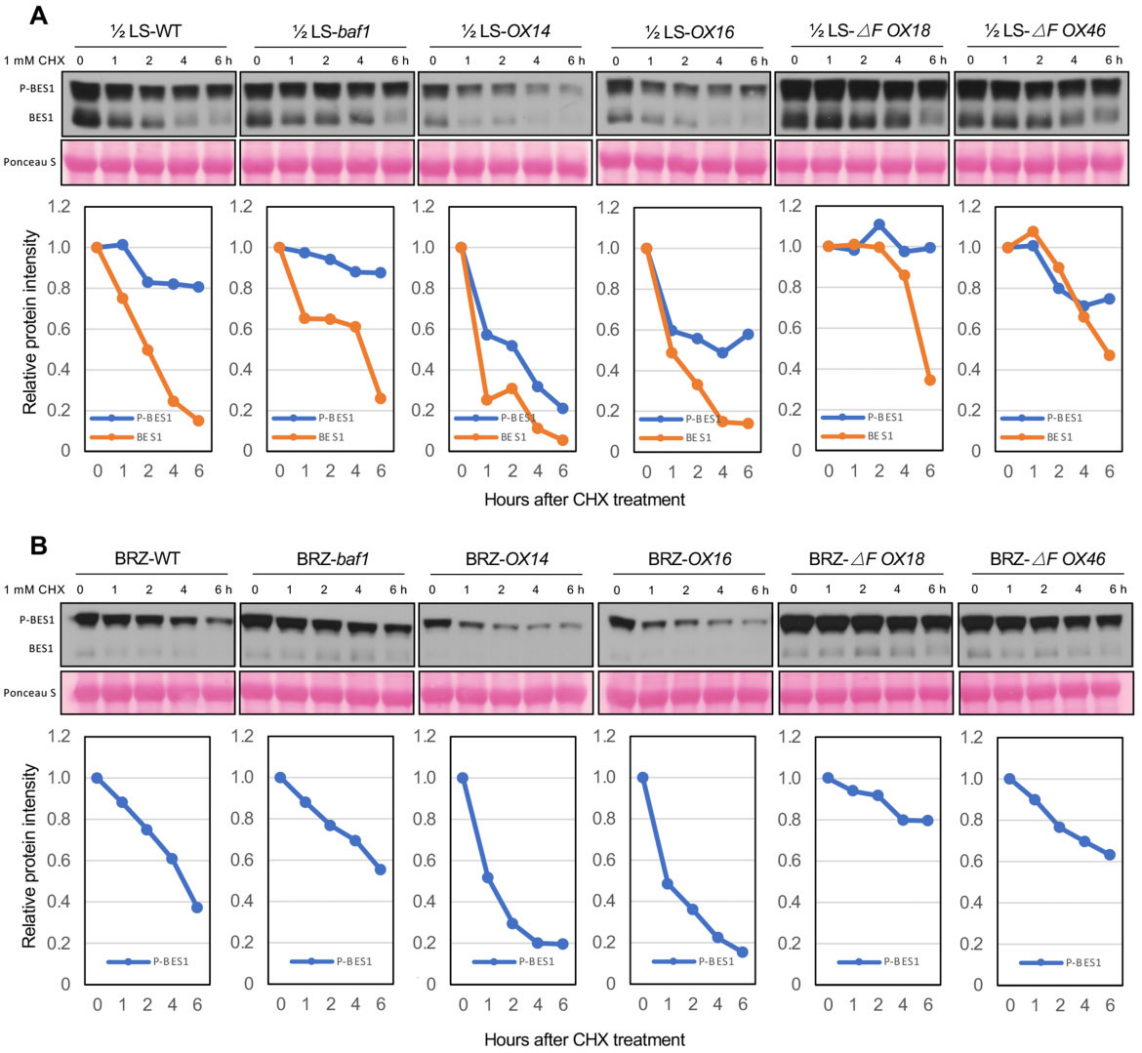
BAF1 mediates BES1 degradation in vivo. A and B, BES1 protein stability was assessed by treating Arabidopsis seedlings grown on 1/2 LS media (A) or 1/2 LS plus 2 µM BRZ (B) with 1 mM CHX for indicated times. WT, *baf1*, *BAF1-FLAG OX* lines, and *BAF1-ΔF-FLAG OX* lines were examined. Samples were analyzed by immunoblotting with anti-BES1 antibody, and relative intensity of protein bands were quantified by ImageJ as shown below each gel. The initial protein levels were defined as 1. Ponceau S staining serves as loading control.

We then assessed BES1 protein stability in vivo in the presence of the protein synthesis inhibitor, cycloheximide (CHX) to estimate the half-life of BES1 protein under control or BR biosynthesis inhibitor BRZ treatment. Levels of both phosphorylated and dephosphorylated BES1 decreased upon CHX treatment in WT under control conditions; in contrast, both forms of BES1 were more stable in the *baf1* mutant and two *BAF1-ΔF-FLAG OX* lines, but less stable in the two *BAF1-FLAG OX* lines (Figure 3, A). However, when seedlings were grown on half-strength Linsmaier and Skoog (1/2 LS) plates containing 2 µM BRZ for 7 days, following the same CHX treatment, most of the BES1 protein was phosphorylated and its degradation was faster than that from the 1/2 LS control. Under this condition, phosphorylated BES1 was strongly stabilized in *baf1* mutant and two *BAF1-ΔF-FLAG OX* lines while destabilized in *BAF1-FLAG OX* lines compared with WT (Figure 3, B).

Using the same samples, we detected INTERACT-WITH-SPT6 (IWS1), a transcription elongation factor that interacts with BES1 (Li et al., 2010), as a negative control in this protein stability assay. IWS1 protein was relatively stable during the same treatments, with no significant differences between genotypes, indicating that BAF1 did not affect the stability of the BES1 coactivator (Supplemental Figure S4). The in vivo BES1 degradation assay strongly supports that BAF1 mediates BES1 degradation, especially when BR levels are reduced upon BRZ treatment.

### BR-regulated growth is altered in *baf1* mutants and *BAF1* overexpression plants

**Figure 4:**
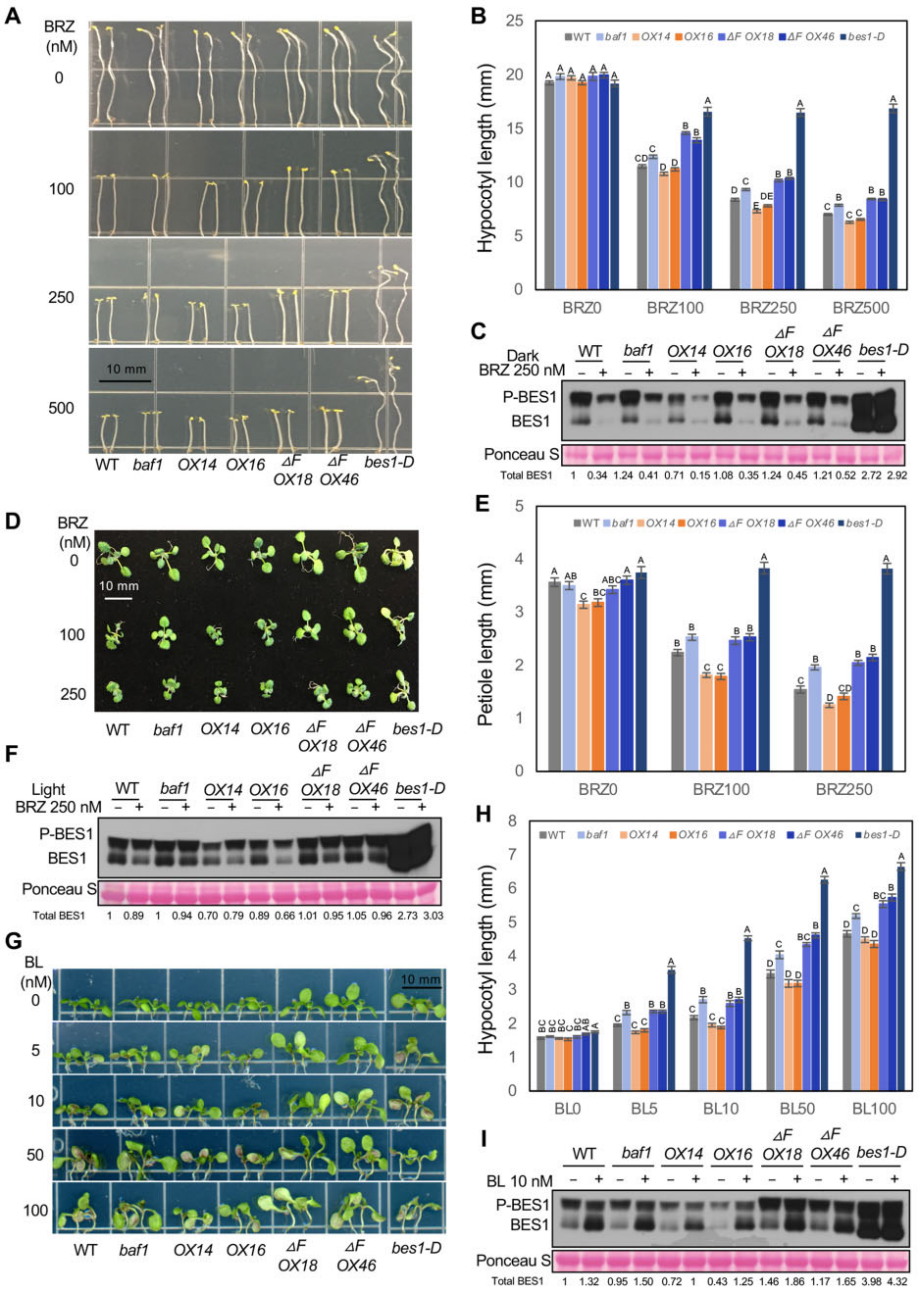
BAF1 negatively regulates BES1-mediated growth. A and B, BRZ sensitivity assay in the dark. WT, *baf1*, *BAF1-FLAG OX* lines, and *BAF1-ΔF-FLAG OX* lines were examined. Seedlings were grown on 1/2 LS medium with different concentrations of BRZ (0, 100, 250, and 500 nM) for 7 days in dark (A). Hypocotyls were measured using ImageJ (B). Data represent mean ± SEM of 24 seedlings from four biological replicates (*n* = 24). C, BES1 protein level from the samples of 250 nM BRZ treatment in dark. Whole seedlings were collected at the end of treatments and analyzed by immunoblotting with anti-BES1 antibody. D and E, BRZ sensitivity assay in light-growing seedlings. Seedlings were grown on 1/2 LS medium with different concentrations of BRZ (0, 100, and 250 nM) for 10 days in continuous light (D). The longest petiole per plant was measured using a ruler (E). Data represent mean ± SEM of 28 seedlings from four biological replicates (*n* = 28). F, BES1 protein level from the samples of 250 nM BRZ treatment in light. Whole seedlings were collected at the end of treatments and analyzed by immunoblotting with anti-BES1 antibody. G and H, BL response assay in light-growing seedlings. WT, *baf1*, *BAF1-FLAG OX* lines, and *BAF1-ΔF-FLAG OX* lines were examined. Seedlings were grown on 1/2 LS medium with different concentrations of BL (0, 5, 10, 50, and 100 nM) for 7 days in continuous light (G). Hypocotyls were measured using ImageJ (H). Data represent mean ± SEM of 24 seedlings from four biological replicates (*n* = 24). I, BES1 protein level in the 10 nM BL treatment in light. Whole seedlings were collected at the end of treatments and analyzed by immunoblotting with anti-BES1 antibody. BES1 and P-BES1 indicates unphosphorylated and phosphorylated BES1, respectively. Ponceau S staining serves as loading control. Quantified relative band intensity of total BES1 protein was listed below using Image J. Scale bars represent 10 mm. Only significant differences between genotypes within one BRZ or BL treatment were compared, which were indicated by different letters according to one-way ANOVA Tukey’s multiple range tests (*P* < 0.05).

The altered BES1 protein levels and stability in *baf1* mutants and overexpression lines suggested that BR-mediated growth responses might be altered. To test this hypothesis, we assayed the responses of these lines to the BR biosynthesis inhibitor BRZ, which reduces the endogenous level of BRs and causes reduced hypocotyl elongation in the dark (Asami et al., 2000). *bes1-D* is a gain-of-function mutation and displays constitutive BR responses and insensitivity to BRZ (Yin et al., 2002). We included *bes1-D* as a positive control in these assays. Seeds were grown on 1/2 LS + 1% sucrose plates with different concentrations of BRZ (0, 100, 250, and 500 nM) in the dark for 7 days and then hypocotyl lengths were measured. *BAF1-OX14* plants had shorter hypocotyls and were slightly more sensitive to BRZ compared with WT at 250 nM BRZ, while *baf1* mutant and *BAF1-ΔF-OX* plants were less sensitive to BRZ and had significantly longer hypocotyls at 250 nM and 500 nM BRZ, although to a lesser extent than *bes1-D* controls (Figure 4, A and B).

To investigate whether the observed phenotypes in different genotypes were related to BES1 protein level, we performed immunoblotting on samples grown at 250 nM BRZ and found that the endogenous BES1 protein level was decreased in *BAF1-OX14* line, while BES1 protein was present at relatively higher levels in *baf1* mutant and *BAF1-ΔF-OX* plants under both control and 250 nM BRZ conditions (Figure 4, C). Since the difference between WT and *BAF1-OX16* line was relatively small in both hypocotyl length and BES1 protein level, we identified two additional lines to confirm (*OX25* and *OX26*, Supplemental Figures S1, C, S5). Both lines had significant shorter hypocotyls and less BES1 protein than WT under control and BRZ-treated conditions (Supplemental Figure S5).

We next examined the BRZ response in light-grown plants by measuring the longest leaf petiole length of each plant. Similarly, *BAF1-OX* plants had significantly shorter leaf petioles at 100 nM BRZ, while *baf1* mutant and *BAF1-ΔF-OX* plants had significantly longer leaf petioles at 250 nM BRZ, compared with WT (Figure 4, D and E). At 250 nM BRZ, the endogenous BES1 protein level was less in *BAF1-OX* lines, but more in *baf1* mutant and *BAF1-ΔF-OX* plants compared with WT (Figure 4, F). *BAF1-OX* plants also had less BES1 protein than WT in control conditions (Figure 4, F). These results indicate that BAF1 negatively modulates BR-regulated growth by reducing BES1 protein levels.

Lastly, we performed BR response assays using BL (the most active BR) by measuring hypocotyl elongation in the light. *baf1* mutant and *BAF1-ΔF-OX* lines were more sensitive to BL and had significant longer hypocotyl lengths than WT after BL treatments (Figure 4, G and H). At 10 nM BL, *baf1* and *BAF1-ΔF-OX* lines also accumulated more BES1 protein than WT (Figure 4, I). *BAF1-OX* lines showed similar BL responses to WT (Figure 4, G and H), even though they had slightly less BES1 protein at 10 nM BL (Figure 4, I), which likely was not sufficient to cause a reduction in hypocotyl elongation in the light. These results further support BAF1 being a negative regulator of BES1 and BR-regulated growth.

### BES1 is negatively regulated by BAF1 and acts downstream of BAF1

**Figure 5:**
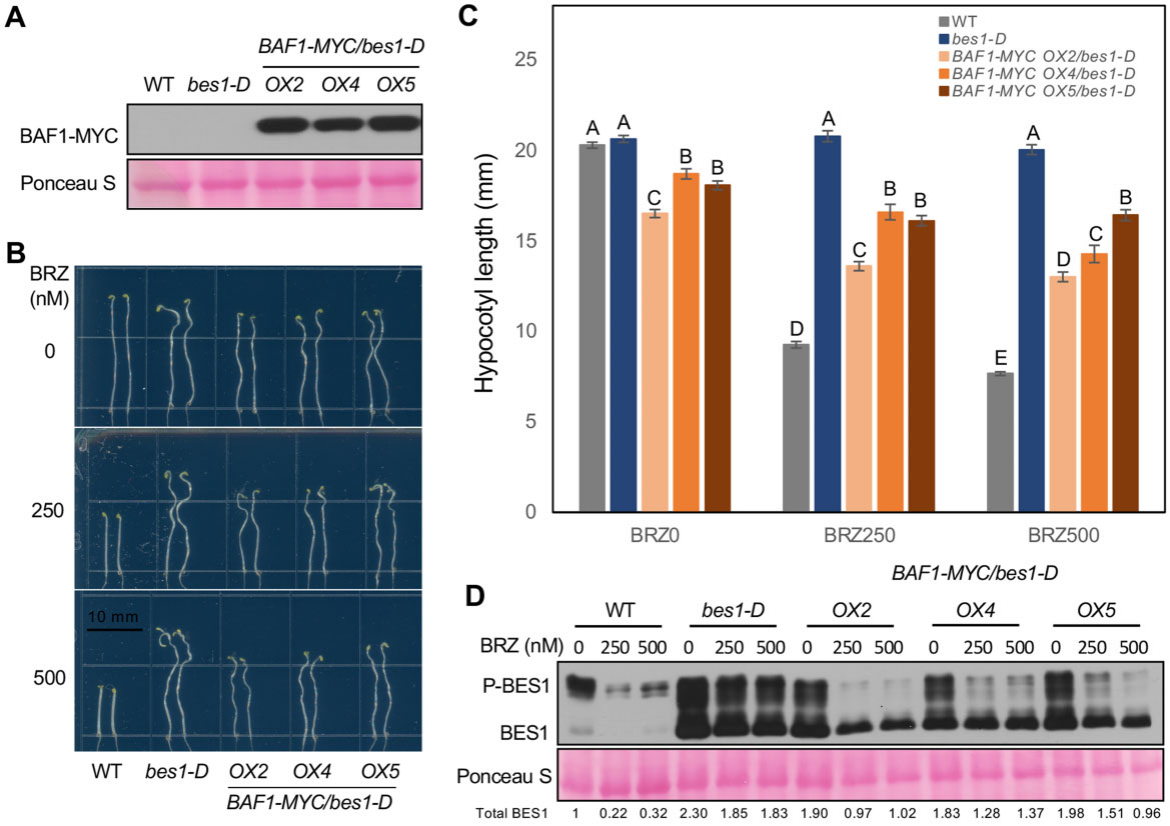
BES1 functions genetically downstream of BAF1. A, Identification of *BAF1-MYC* overexpressing lines in *bes1-D* background by immunoblotting against anti-MYC (rabbit) antibody. Three T3 homozygous lines were used. Ponceau S staining serves as loading control. B and C, BRZ sensitivity assay in the dark. WT, *bes1-D*, and three *BAF1-MYC OX* lines in *bes1-D* were examined. Seedlings were grown on 1/2 LS medium with different concentrations of BRZ (0, 250, and 500 nM) for 7 days in dark (B). Hypocotyls were measured using ImageJ (C). Data represent mean ± SEM of 24 seedlings from four biological replicates (*n* = 24). Only significant differences between genotypes within one BRZ treatment were compared, which were indicated by different letters according to one-way ANOVA Tukey’s multiple range tests (*P* < 0.05). Scale bar represents 10 mm. D, BES1 protein level from the samples of treatments in (B). Whole seedlings were collected at the end of treatments and analyzed by immunoblotting with anti-BES1 antibody. BES1 and P-BES1 indicates unphosphorylated and phosphorylated BES1, respectively. Ponceau S staining serves as loading control. Quantified relative band intensity of total BES1 protein was listed below using Image J.

To further verify that BES1 is negatively regulated by BAF1 and acts downstream of BAF1, we overexpressed *35S:BAF1-MYC* in *bes1-D* and obtained T3 homozygous lines with stable BAF1 expression (Figure 5, A). Phenotypic analysis showed that *BAF1* overexpression significantly increased the sensitivity of *bes1-D* to BRZ. Hypocotyl lengths and BES1 protein levels in three *BAF1-MYC* overexpression lines were significantly reduced compared with *bes1-D* under both control and BRZ treatments, although they still displayed significantly longer hypocotyl and more BES1 than WT (Figure 5, B–D). These results demonstrated that BES1 is negatively regulated by BAF1 and acts downstream of BAF1.

SINATs and MAX2 E3 ligases are involved in BES1 degradation (Wang et al., 2013; Nolan et al., 2017; Yang et al., 2017). To determine the relationship between BAF1 and SINATs as well as BAF1 and MAX2, we generated *baf1 SINAT RNAi* and *baf1 max2* double mutants. We treated these mutants with BRZ in either dark or relatively weak light conditions and measured their hypocotyl lengths. In the dark, the BRZ response of *baf1 SINAT RNAi* was like that of the *baf1* mutant since *SINAT RNAi* was significantly more sensitive than WT (Supplemental Figures S6, A and B). SINATs control the light-mediated stability of BES1 (Yang et al., 2017) and likely have other substrates in the dark contributing to hypocotyl elongation. However, *baf1 max2* double mutant displayed significant longer hypocotyl than either single mutant or WT under each BRZ concentration (Supplemental Figure S6, A and B), indicating BAF1 and MAX2 function redundantly in BRZ response in the dark. In the light, *SINAT RNAi* displayed longer hypocotyls than WT as expected, and *baf1 SINAT RNAi* had much longer hypocotyls than *baf1* and *SINAT RNAi* under both control and BRZ treatment (Supplemental Figure S6, C and D). *max2* also had longer hypocotyls than WT, and *baf1 max2* only exhibited longer hypocotyls than *baf1* and *max2* under BRZ treatment (Supplemental Figure S6, C and D). This result indicated that BAF1 functions redundantly with SINATs and MAX2 in BRZ response mostly in the light. We also immunoprecipitated BES1 from WT, *baf1*, *SINAT RNAi*, and *baf1 SINAT RNAi*, and found that there was less ubiquitinated BES1 in *baf1 SINAT RNAi* than either *baf1* or *SINAT RNAi*, indicating that BAF1 and SINATs also function redundantly in BES1 ubiquitination in the light (Supplemental Figure S6, E).

### Differentially expressed genes in *baf1* significantly overlap with BES1-D-regulated and BRZ-regulated genes

**Figure 6:**
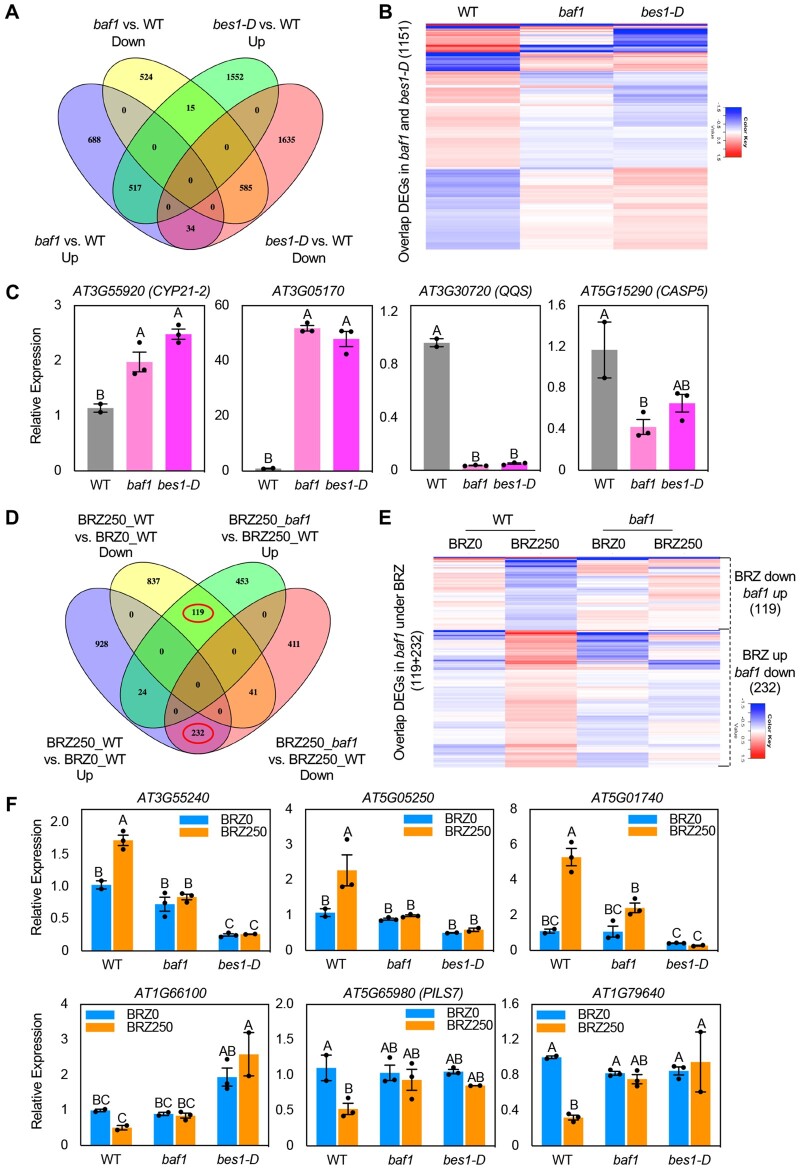
BAF1 regulation of BES1-D regulated genes and BRZ-responsive genes. A, Venn diagrams showing overlap in DEGs of *baf1* and *bes1-D* from QuantSeq. B, Clustering analysis of overlapped DEGs from (A). The color legend indicates normalized gene expression value among genotypes. C, RT-qPCR validation of selected genes from (B). The relative gene expression was normalized to the expression of the reference gene *ACTIN2*. RT-qPCR was performed on three technical replicates of three independent biological replicates for *baf1* and *bes1-D* and 2 for WT samples. D, Venn diagrams showing overlap among BRZ-responsive genes and DEGs in *baf1* under BRZ treatment from QuantSeq. Red circle indicates genes differentially regulated by BRZ and BAF1. E, Clustering analysis of overlapped DEGs in red circle from (C). The color legend indicates normalized gene expression value among genotypes and treatments. F, RT-qPCR validation of selected genes from (E). The relative gene expression was normalized to the expression of the reference gene *ACTIN2*. RT-qPCR was performed on three technical replicates of two to three independent biological replicates. Data represent mean ± SEM. Different letters indicate significant difference according to one-way ANOVA Tukey’s multiple range tests (*P* < 0.05).

To compare the genes that are differentially expressed in *baf1* and *bes1-D*, we isolated total RNA from 7-day-old whole seedlings and subjected them to 3′-based RNA sequencing (Quantseq) analysis. We identified 2,363 and 4,338 differentially expressed genes (DEGs, fold change > 1.1, *P*-value < 0.05) in *baf1* and *bes1-D* compared with WT, respectively (Figure 6, A andSupplemental Data Set S1). A total of 1,151 genes of the 2,363 DEGs identified from *baf1* were also regulated by *bes1-D* (Figure 6, A). Clustering analysis of these genes showed that 1,102 genes (95.7%) were regulated in the same manner as those in *bes1-D* (Figure 6, B), that is BES1-D up-regulated genes are up-regulated in *baf1* and BES1-D down-regulated genes are down-regulated in *baf1*, although to a lesser extent than in *bes1-D*. Of the 551 genes upregulated and the 600 genes downregulated in *baf1*, 517 (93.8%, *P*-value = 2.06*E*−298) and 585 (97.5%, *P*-value = 2.71*E*−389) genes were up- and downregulated in *bes1-D*, respectively (Figure 6, A and B andSupplemental Data Set S1). From these two overlapped groups, we picked two upregulated genes (*CYP21-2* and *AT3G05170*) and two downregulated genes (*QQS* and *CASP5*), and verified the results by RT-qPCR (Figure 6, C).

We also treated WT and *baf1* with 250 nM BRZ for 7 days and performed Quantseq transcriptomic analysis. We found 2,181 DEGs (fold change > 1.1, *P*-value < 0.05) that were regulated by BRZ treatment in WT, of which 1,184 genes were upregulated and 997 genes downregulated by BRZ (Figure 6, D andSupplemental Data Set S1). Under BRZ treatment, we identified 1,280 genes that were differently expressed in the *baf1* mutant compared with WT (Figure 6, D andSupplemental Data Set S1; fold change > 1.1, *P*-value < 0.05). A total of 119 (*P*-value = 1.25*E*−61) out of 596 BRZ-upregulated genes and 232 (*P*-value = 6.54*E*−162) of 684 BRZ-downregulated genes in *baf1* overlapped with BRZ-downregulated and -upregulated genes in WT, respectively (Figure 6, D). Clustering analysis of these genes showed that in general BRZ-repressed genes (i.e. BR-induced genes) were up-regulated in *baf1* and BRZ-induced genes (i.e. BR-repressed genes) were down-regulated in *baf1* under BRZ treatment (Figure 6, E). We repeated the same treatment in WT and *baf1*, with *bes1-D* as control, and performed RT-qPCR analysis of three BRZ-induced genes and three BRZ-repressed genes (Figure 6, F) and confirmed the results from Quantseq. Taken together, our global gene expression studies demonstrated that significant numbers of BR- or BES1-induced genes are up-regulated in *baf1* and BR- or BES1-repressed genes are down-regulated in the mutant, supporting the hypothesis that BAF1 is a negative regulator of BR-regulated gene expression.

### BAF1 targets BES1 for autophagic degradation

Previously BES1 was reported to be degraded through both autophagy and proteasome pathways (Nolan et al., 2017). The two most abundant ubiquitin attachments, Lys48 and Lys63 linkages, were both found in BES1 ubiquitination in *BAF1* overexpression plants (Figure 2, F). Lys48 linkage usually serves as a strong proteasomal degron while Lys63-linked chains often function as autophagic degrons (Ji and Kwon, 2017). These results prompted us to assess the pathways by which BAF1 mediates BES1 degradation. We first treated plants with the proteasome inhibitor MG132 or the protease inhibitor E64d, which can block vacuolar degradation during autophagy (Klionsky et al., 2021). We tested BES1 protein stability in WT and *BAF1 OX14* seedlings treated with CHX with or without MG132 or E64d. In both WT and *BAF1 OX14*, BES1 protein degradation could be reduced by either MG132 or E64d (Supplemental Figure S7, A), suggesting both proteasomal and autophagic degradation pathways are responsible for BES1 stability in planta. Then we transiently expressed BES1 and BAF1 in *N. benthamiana*, followed by treatment with E64d or MG132. As expected, BES1 protein accumulated in the presence of either inhibitor when co-expressed with a control construct, as previously reported (Nolan et al., 2017).

**Figure 7:**
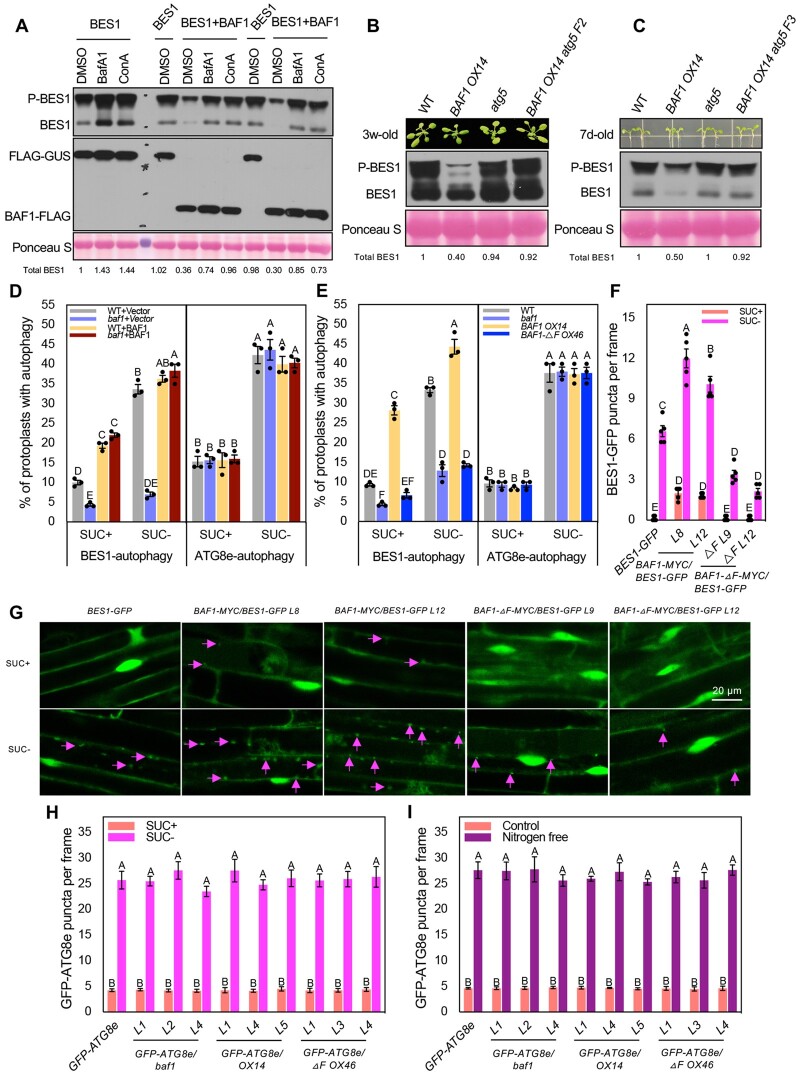
BAF1 mediates BES1 degradation through autophagy. A, Responses of BAF1-mediated BES1 degradation to autophagy inhibitors in *N. benthamiana*. BES1 was co-infiltrated with BAF1-FLAG or control vector into *N. benthamiana*. After 12 h, DMSO, 1 µM ConA or 2 µM BafA1 were infiltrated into the same leaf area as infiltrated before. For comparison, combinations were infiltrated into the same leaf to reduce variation. Two to three leaves for each combination were infiltrated as biological replicates. Samples were collected 24 h after addition of inhibitors and analyzed by immunoblotting with anti-BES1 and anti-FLAG (rabbit) antibodies. Ponceau S staining serves as protein loading control. Quantified relative band intensity of total BES1 protein was listed below using Image J. B and C, BES1 level in WT, *BAF1 OX14*, *atg5*, and *BAF1 OX14 atg5* double mutants. 3-week-old adult 5th leaf (B) or 7-day-old seedlings (C) were collected and analyzed by immunoblotting with anti-BES1 antibody. Ponceau S staining serves as loading control. Quantified relative band intensity of total BES1 protein was listed below using Image J. D and E, Quantification of protoplasts with BES1 selective autophagy and ATG8e-labeled bulk autophagy. D, BES1-GFP and mCherry-ATG8e were separately transformed into WT or *baf1* Arabidopsis protoplasts. BAF1-FLAG was co-transformed for complementation. E, BES1-GFP and mCherry-ATG8e were separately transformed into WT, *baf1*, *BAF1 OX14*, and *BAF1-ΔF OX46* protoplasts. Protoplasts were treated without or with 0.5% (w/v) sucrose for 36 h before microscopy. Protoplasts with more than three visible autophagosomes were counted as active for autophagy. A total of 100 protoplasts were observed per treatment per genotype, and the percentage of protoplasts with active autophagy was calculated and averaged from three independent experimental replicates. Data represent mean ± SEM (*n* = 3). Only significant differences between bars within BES1-autophagy or ATG8e-autophagy were compared, which were indicated by different letters according to one-way ANOVA Tukey’s multiple range tests (*P* < 0.05). F, BES1-GFP labeled puncta numbers in the Arabidopsis roots. *35S:BES1-GFP* transgenic plants as control, two homozygous lines of *35S:BAF1-MYC* overexpressing or *35S:BAF1-ΔF-MYC* in *35S:BES1-GFP* background were examined. Seven-day-old seedlings were transferred to 1/2 LS liquid media with or without sucrose for 16 h. Three to five representative images in the root elongation zone were photographed per seedling and the number of BES-GFP puncta in each image was counted and averaged. A total of five seedlings were observed per treatment. Data represent mean ± SEM (*n* = 5). Different letters indicate significant difference according to one-way ANOVA Tukey’s multiple range tests (*P* < 0.05). G, Confocal images of BES1-GFP puncta in the roots of (F). GFP fluorescent signals were collected with excitation and emission at 488 and 555 nm, respectively. Magenta arrows indicate the puncta. Scale bar represents 20 µm. H and I, GFP-ATG8e labeled autophagosomes in response to sucrose starvation (H) or nitrogen starvation (I). GFP-ATG8e and three individual T2 lines from either *baf1*, *BAF1 OX14*, or *BAF1-ΔF OX46* backgrounds were examined. GFP-ATG8e labeled autophagosomes in the roots were observed by epifluorescence microscopy using a GFP filter. Two to three images in the root elongation zone were photographed per seedling and the number of GFP-ATG8e puncta in each image was counted and averaged. A total of ten seedlings were observed per treatment. Data represent mean ± SEM (*n* = 10). Different letters indicate significant difference according to one-way ANOVA Tukey’s multiple range tests (*P* < 0.05).

However, while co-expression of BES1 and BAF1 led to a reduction in BES1 level in the control treatment, E64d significantly blocked BAF1-mediated BES1 degradation, while MG132 only had a small effect (Supplemental Figure S7, B). We further confirmed this observation by introducing two more autophagy-related inhibitors, Bafilomycin A1 (BafA1) and Concanamycin A (ConA), both of which are inhibitors of V-type ATPase and prevent the degradation of autophagosomes in the vacuoles or lysosomes (Klionsky et al., 2021). Both BafA1 and ConA blocked BES1 degradation by BAF1 transient expression in *N. benthamiana* (Figure 7, A). The results suggested that BAF1 mediates BES1 protein degradation preferentially through autophagy at least under certain conditions.

To genetically confirm that BAF1-mediated BES1 ubiquitination targets BES1 to the autophagy pathway, we constructed a *BAF1 OX14 atg5* double mutant and analyzed the effect on BES1 accumulation. Strikingly, *atg5* suppressed *BAF1* overexpression phenotype in BES1 accumulation in both adult plants and seedlings (Figure 7, B and C), indicating that ATG5 acts downstream of BAF1 in targeting BES1 to the autophagy pathway.

### BAF1 mediates selective autophagy of BES1 during sucrose starvation

Autophagy is strongly induced under nutrient-limiting conditions, including fixed-carbon starvation. Previously we found that *bes1-D*, which displays constitutive BR response and increased BES1 levels, was more sensitive to fixed-carbon starvation (Nolan et al., 2017). From our published RNA-seq dataset for fixed-carbon starvation (Nolan et al., 2017), we found that *BAF1* transcripts are significantly induced by approximately four-fold by fixed-carbon starvation in either WT or *bes1-D* plants (Supplemental Figure S9, A). We hypothesized that BAF1 targets BES1 for degradation under sucrose starvation conditions. To test this idea, we examined autophagy in WT and *baf1* mutant protoplasts subjected to sucrose starvation. BES1-GFP labeled puncta, an indicator of selective autophagy of BES1, were greatly induced by sucrose starvation in WT, and the induction was significantly compromised in the *baf1* mutant. However, *BAF1* overexpression rescued this reduced BES1 autophagy in *baf1* (Figure 7, D). We also tested mCherry-ATG8e labeled bulk autophagy following the same treatments. Sucrose starvation induced bulk autophagy in both WT and *baf1* mutant with no significant difference (Figure 7, D).

Similarly, we examined BES1-GFP labeled selective autophagy or mCherry-ATG8e labeled bulk autophagy in protoplasts from different genotypes (WT, *baf1*, *BAF1 OX14*, and *BAF1-ΔF OX46*). There was a significant reduction in the induction of selective BES1 autophagy by sucrose starvation in *baf1* and *BAF1-ΔF OX46* protoplasts. *BAF1 OX14* showed constitutive BES1 autophagy with significantly higher levels compared with WT under both control and sucrose starvation (Figure 7, E). However, there was no significant difference in sucrose starvation-induced bulk autophagy among these genotypes (Figure 7, E). The confocal images represent the colocalization of BES1-GFP and mCherry-ATG8e when co-expressed in the protoplasts of different genotypes. The mCherry-ATG8e puncta were similarly induced by sucrose starvation in each genotype, but BES1-GFP puncta displayed induction difference in different genotypes (Supplemental Figure S8). These results demonstrated that BAF1 is required for selective autophagy of BES1 under sucrose starvation, but not for bulk autophagy.

To further confirm this observation, we generated transgenic plants by overexpressing *35S:BAF1-MYC* and *35S:BAF1-ΔF-MYC* in the background of *35S:BES1-GFP* plants. Two homozygous lines of each construct were selected and immunoblotting showed their expression and effect on BES1-GFP protein. As expected, BES1-GFP protein levels were greatly decreased in *BAF1-MYC* overexpression lines but accumulated in *BAF1-ΔF-MYC* overexpression lines (Supplemental Figure S9, B). Using these lines, we examined BES1-GFP puncta by confocal microscopy after 16 h starvation. In line with the transient expression results, *BAF1-MYC* overexpression lines had significantly higher levels of BES1-GFP puncta than control plants under both control and sucrose starvation, while *BAF1-ΔF-MYC* overexpression lines had fewer BES1-GFP puncta than WT under sucrose starvation (Figure 7, F–G). We also introduced *35S:GFP-ATG8e* into relevant backgrounds (*baf1*, *BAF1 OX14*, and *BAF1-ΔF OX46*) and assessed bulk autophagy induced by sucrose starvation or nitrogen starvation. Consistent with the transient results, GFP-ATG8e labeled bulk autophagy was induced normally by sucrose or nitrogen starvation and there were no significant differences among the different genotypes tested (Figure 7, H and I and Supplemental Figure S9, D). These transgenic plants further confirmed the conclusion that BAF1 is essential for induction of selective BES1 autophagy but not bulk autophagy under sucrose starvation.

We employed long-term sucrose starvation (one-type of fixed-carbon starvation) in plates to test whether the *baf1* mutant is sensitive to starvation, with WT and *bes1-D* as controls. As expected, *bes1-D* was sensitive to sucrose starvation (Nolan et al., 2017; Supplemental Figure S10, A and B). Compared with WT, the *baf1* mutant was significantly more sensitive to sucrose starvation, although not to the extent of *bes1-D* (Supplemental Figure S10, A and B). We then tested the sensitivity of *BAF1 OX14* and *BAF1-ΔF OX46* plants to sucrose starvation. Interestingly, *BAF1 OX14* was highly tolerant of sucrose starvation, while *BAF1-ΔF OX46* was sensitive to the stress, similar to the *baf1* mutant (Supplemental Figure S10, C and D).

We also subjected these lines to nitrogen starvation for 4 days to see whether they have similar responses. As a control, *atg5* was sensitive to nitrogen starvation as expected. However, there were no obvious differences among WT, *baf1*, *BAF1 OX14*, *BAF1-ΔF OX46*, and *bes1-D* (Supplemental Figure S10, E). We also did not observe BES1-GFP puncta upon nitrogen starvation in either control plants or *BAF1-MYC* or *BAF1-ΔF-MYC* overexpression lines (Supplemental Figure S9, C). Therefore, BAF1-mediated selective BES1 autophagy is specific to carbon starvation but not nitrogen starvation.

Lastly, we examined how sucrose starvation sensitivity is related to BES1 level. We performed sucrose starvation in the dark and assessed BES1 protein level. Seven-day-old seedlings were transferred to both control and sucrose-free plates and grown in the dark for 3 days. In WT seedlings, BES1 protein level decreased by more than half after growth in the dark for 3 days with sucrose, and it decreased further in the absence of sucrose. However, this decrease was much less in *baf1* mutants (Supplemental Figure S10, F). These results suggest that the sensitivity to sucrose starvation of the *baf1* mutant is correlated with increased BES1 protein levels, which is in line with the observation that BES1 plays a negative role in survival during sucrose starvation.

### BAF1-meditated selective autophagy of BES1 is dependent on DSK2

**Figure 8:**
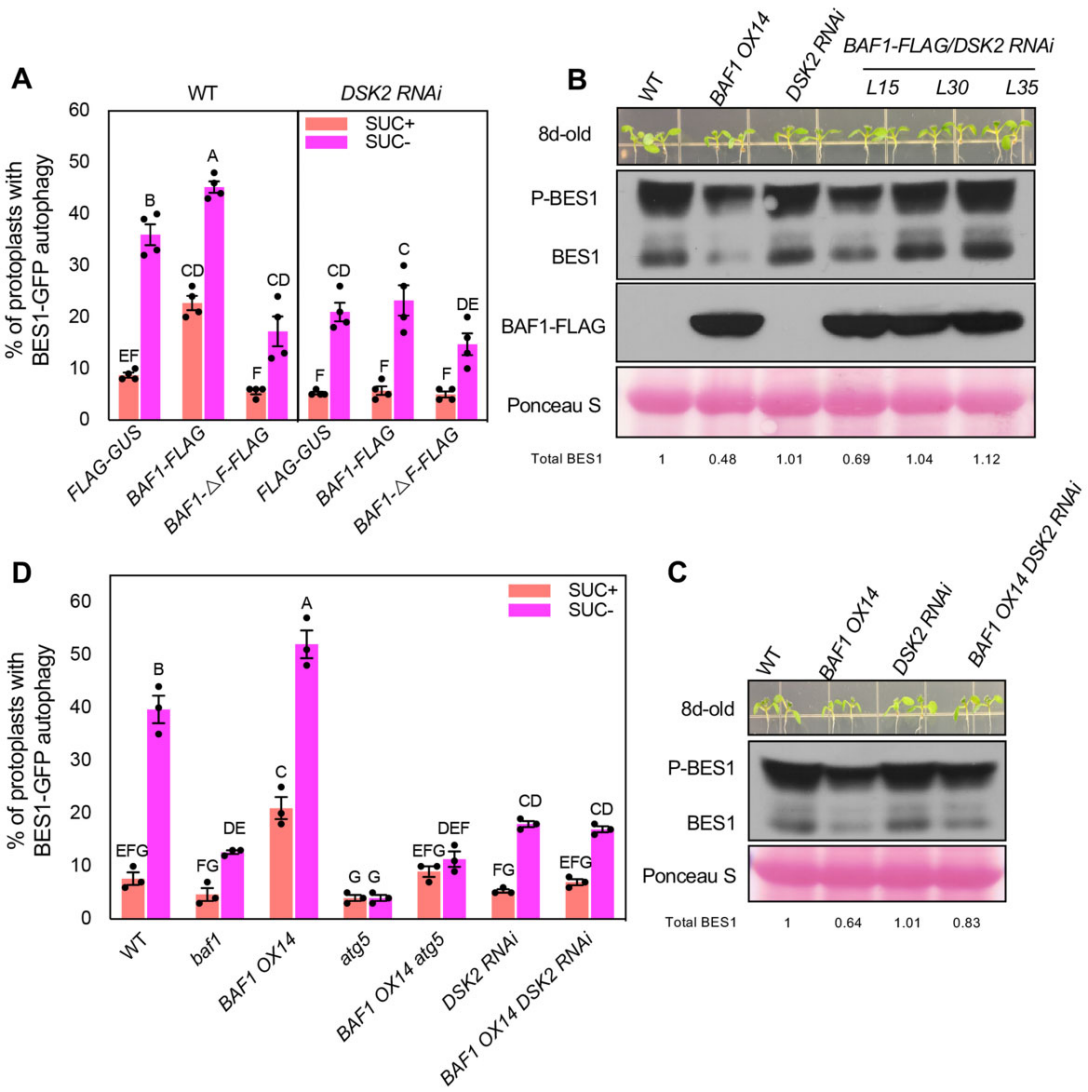
BAF1-mediated selective BES1 autophagy is dependent on DSK2. A, Quantification of protoplasts with BES1 selective autophagy in WT and *DSK2 RNAi* protoplasts. BES1-GFP was co-transformed with BAF1-FLAG or BAF1-ΔF-FLAG or control vector into WT and *DSK2 RNAi* protoplasts. Protoplasts were treated without or with 0.5% (w/v) sucrose for 36 h before microscopy. Protoplasts with more than three visible autophagosomes were counted as active for autophagy. A total of 100 protoplasts were observed per treatment per genotype, and the percentage of protoplasts with active autophagy was calculated and averaged from four independent experimental replicates. Data represent mean ± SEM (*n* = 4). B, BES1 protein level in BAF1-FLAG overexpressing T2 lines in the background of *DSK2 RNAi*. Eight-day-old seedlings were collected and analyzed by immunoblotting with anti-FLAG (rabbit) and anti-BES1 antibodies. C, BES1 protein level in WT, *BAF1 OX14*, *DSK2 RNAi*, and *BAF1 OX14 DSK2 RNAi F2* plants. Eight-day-old seedlings were collected and analyzed by immunoblotting with anti-BES1 antibody. Ponceau S staining serves as loading control. Quantified relative band intensity of total BES1 protein was listed below using Image J. D, Quantification of protoplasts with BES1 selective autophagy in *BAF1 OX14 atg5* and *BAF1 OX14 DSK2 RNAi* double mutants and control mutants. *BES1-GFP* was transformed into protoplasts from different genotypes. Protoplasts were treated without or with 0.5% (w/v) sucrose for 36 h before observation under microscopy. A total of 100 protoplasts were observed per treatment per genotype, and the percentage of protoplasts with active autophagy was calculated and averaged from three independent experimental replicates. Data represent mean ± SEM (*n* = 3). Different letters indicate significant difference according to one-way ANOVA Tukey’s multiple range tests (*P* < 0.05).

We have previously established that the autophagy receptor DSK2 targets BES1 to selective autophagy under sucrose starvation conditions (Nolan et al., 2017). To explore if BAF1-mediated selective autophagy of BES1 is dependent on DSK2, we firstly compared selective BES1 autophagy in WT and *DSK2 RNAi* protoplasts upon transient expression of BAF1. BES1 autophagy induction by sucrose starvation was significantly reduced in *DSK2 RNAi* when compared with that in WT (Figure 8, A), which is consistent with previous findings (Nolan et al., 2017). Overexpressing *BAF1* greatly increased BES1 autophagy in WT under both control and sucrose starvation compared with the control construct, and this effect was abolished in *DSK2 RNAi* protoplasts (Figure 8, A). This result suggests that BAF1-mediated BES1 autophagy at least partially depends on DSK2 function.

We then transformed *35S:BAF1-FLAG* into *DSK2 RNAi* plants and identified three lines with similar BAF1 protein amount as *BAF1 OX14* (Figure 8, B). The BES1 level in these three lines was more than that in *BAF1 OX14* plants, indicating *DSK2 RNAi* suppressed *BAF1 OX14* phenotype in BES1 accumulation (Figure 8, B). We also generated a *BAF1 OX14 DSK2 RNAi* double mutant, in which there was relatively more BES1 protein than that in *BAF1 OX14* plants (Figure 8, C). These results suggested that BAF1-mediated selective autophagy of BES1 for degradation is dependent on DSK2.

**Figure 9:**
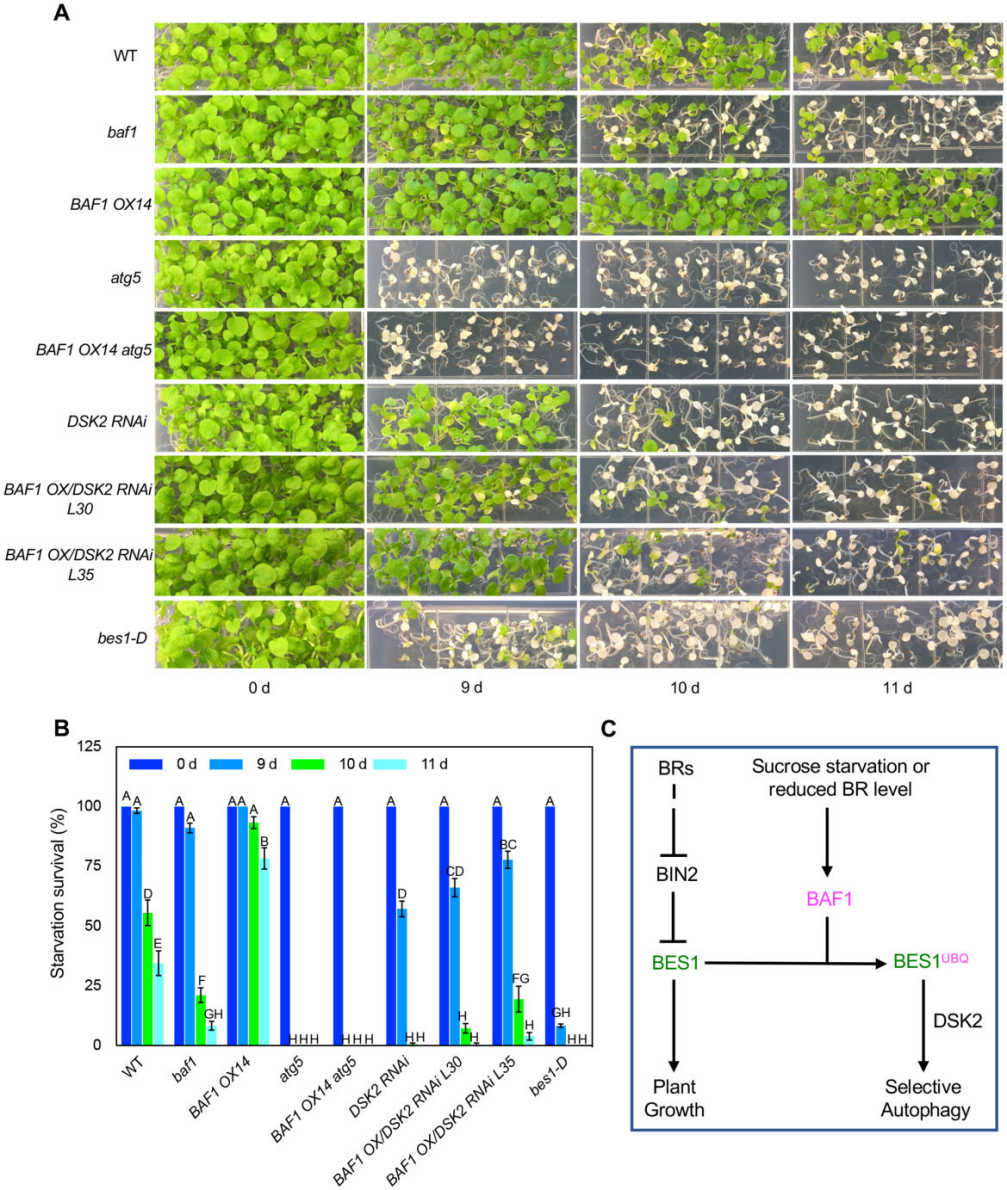
BAF1-mediated plant sensitivity to long-term sucrose starvation is dependent on ATG5 and DSK2. A and B, Seedling phenotype and survival percentage after long-term sucrose starvation in the dark (with indicated times) followed by 7 days recovery under the light. Seedlings remaining green or with new growth emerging are considered as surviving. Data represent mean ± SEM from six biological replicates (*n* = 6); each replicate contains 30 seedlings. Different letters indicate significant difference according to one-way ANOVA Tukey’s multiple range tests (*P* < 0.05). C, A model for BAF1 function in BES1 degradation. Under normal growth condition, BRs function to induce BES1 to promote plant growth. Under sucrose starvation or reduced BR conditions, E3 ubiquitin ligase BAF1 ubiquitinates BES1 and targets it to selective autophagy through ubiquitin receptor DSK2 to slow down plant growth.

Finally, we isolated protoplasts from double mutants *BAF1 OX14 atg5* and *BAF1 OX14 DSK2 RNAi* and tested selective BES1 autophagy upon sucrose starvation. The results showed very clearly that the increased BES1-autophagy phenotype in *BAF1 OX14* protoplasts was suppressed in *atg5* or *DSK2 RNAi* background (Figure 8, D). We then subjected these double mutants including all the control mutants to long-term sucrose starvation assays. As expected, the high tolerance to sucrose starvation in *BAF1 OX14* was almost abolished in *BAF1 OX14 atg5* double mutant and *BAF1 OX/DSK2 RNAi* lines, which became as sensitive as *atg5* and *DSK2 RNAi* mutants, respectively (Figure 9, A and B). Together these genetic data further strongly demonstrated that BAF1-mediated selective autophagy of BES1 is dependent on ATG5 and DSK2.

## Discussion

Plants face ever-changing environmental conditions and have to integrate endogenous hormonal signals with various environmental stimuli to maintain an appropriate balance between growth and stress responses. Understanding the molecular mechanisms that coordinate growth and stress responses is critical for improving plant productivity and survival under these challenging conditions. BES1 and BZR1 are emerging as hubs coordinating BR-mediated growth and stress responses through several reported mechanisms (Chen et al., 2017; Nolan et al., 2017; Ye et al., 2017; Jiang et al., 2019; Xie et al., 2019). Multiple post-translational modifications modulate BES1 and BZR1 functions, including phosphorylation, ubiquitination, sumoylation, and oxidation (Tian et al., 2018; Zhang et al., 2019; Nolan et al., 2020; Srivastava et al., 2020).

Several distinct E3 ubiquitin ligases ubiquitinate and facilitate BES1 and/or BZR1 protein degradation in tissue-specific or developmental stage-specific pathways, or under different hormonal and environmental conditions, involving either the proteasome or autophagy (Yang and Wang, 2017; Kim et al., 2019; Nolan et al., 2020). In this study, we identified an F-box family E3 ubiquitin ligase BAF1 and provided evidence that BAF1 mediates BES1 degradation under sucrose starvation or reduced BR conditions.

Multiple lines of evidence support the idea that BAF1 ubiquitinates BES1 and leads to its degradation. First, BAF1 interacts with BES1 both in vitro and in vivo (Figure 1). Second, BAF1 overexpression leads to BES1 ubiquitination and degradation in vivo (Figures 2, 3); Third, BES1 is stabilized in the *baf1* mutant and *BAF1-ΔF* dominant-negative transgenic lines and, on the other hand, is destabilized in *BAF1* over-expression lines (Figure 3).

Consistent with its role as an E3 ubiquitin ligase for BES1, BAF1 functions as a negative regulator for BR-regulated gene expression and BR responses. A loss-of-function *baf1* mutant had up-regulation of BES1-induced genes (and down-regulation of BES1-repressed genes) and displayed increased BR-responsive phenotypes including tolerance to the BR biosynthesis inhibitor BRZ. *BAF1-ΔF* transgenic plants had even stronger tolerance to BRZ and were more responsive to BL in hypocotyl elongation assays, while *BAF1* overexpression lines showed the opposite responses (Figures 4–6). Our genetic analysis also demonstrated that BES1 acts downstream of BAF1 in mediating BR-regulated growth (Figure 5).

The fact that BAF1 mutants did not have obvious phenotypes under normal growth conditions suggests that BAF1 may function during stress to slow down plant growth by ubiquitinating BES1 for degradation. Previous global gene expression data showed that *BAF1* was transcriptionally induced by fixed-carbon starvation (Nolan et al., 2017), indicating that BAF1 might be involved in sucrose starvation. Indeed, *BAF1* overexpression plants exhibited a strong tolerance to sucrose starvation, while the *baf1* mutant and *BAF1-ΔF* dominant-negative transgenic lines were more sensitive to this stress (Supplemental Figure S10 and Figure 9).

Our results indicated that BAF1 targets BES1 to the selective autophagy pathway under sucrose starvation. Most of the reported E3 ubiquitin ligases mediate BES1 degradation through the 26S proteasomal pathway (Wang et al., 2013; Kim et al., 2014, 2019; Yang et al., 2017). BES1 can also be degraded by selective autophagy via the ubiquitin receptor protein DSK2 (Nolan et al., 2017). Several lines of evidence demonstrated that BAF1 ubiquitinated BES1 can be targeted to selective autophagy under sucrose starvation. First, BAF1 can ubiquitinate BES1 through both K63- and K48-linked ubiquitination (Figure 2). Usually, K48 linkage is the most abundant linkage in plants and serves as a strong proteasomal degron and K63-linked chains function as general autophagic degrons (Kim et al., 2013; Ji and Kwon, 2017). One study reveals that K63 ubiquitination could also trigger proteasomal degradation by seeding branched ubiquitin chains, demonstrating a ubiquitin ligase can catalyze both K48 and K63 linkages (Ohtake et al., 2018). Indeed, we found that autophagy and proteasome inhibitors can stabilize BAF1-mediated degradation of BES1 in Arabidopsis (Supplemental Figure S7, A and Figure 7, A), suggesting that BAF1 ubiquitinated BES1 can be degraded through either autophagy or proteasome degradation pathways. Second, autophagy inhibitors but not MG132 strongly blocked BAF1-mediated degradation of BES1 when transiently co-expressed in *N. benthamiana* (Supplemental Figure S7, B and Figure 7, A). Genetics further showed that BAF1-mediated degradation and selective autophagy of BES1 and tolerance to sucrose starvation were compromised in an *atg5* mutant that abolishes autophagy (Figures 7, B and C, 8, D, and 9), supporting the conclusion that BAF1-ubiquitinated BES1 can be preferentially targeted to autophagy under sucrose starvation. Third, selective autophagy of BES1 during sucrose starvation was greatly compromised in *baf1* and *BAF1-ΔF* overexpression plants, but autophagy of BES1 was up-regulated by *BAF1* overexpression even in control conditions (Figure 7, D–G). However, BAF1 did not affect bulk autophagy induced by either sucrose starvation or nitrogen starvation (Figure 7, H–I). Finally, BAF1-mediated selective autophagy degradation of BES1 is dependent on ubiquitin receptor DSK2 (Figures 8, 9). DSK2 was reported as an ubiquitin receptor responsible for selective BES1 autophagy under sucrose starvation (Nolan et al., 2017). It is worth noting that BAF1 ubiquitinated BES1 can also be targeted to proteasomal degradation (Supplemental Figure S7). How the selective autophagy and proteasome pathways coordinate in degrading BES1 under what specific conditions remain to be determined.

What is the biological function of BAF1? Energy availability needs to be maintained for plant growth and survival. When energy availability is perturbed by conditions interfering with carbon assimilation or utilization, such as short-day photoperiod, extended darkness, shading, and drought, plants experience a transient or long-term sugar starvation, leading to the interruption of plant growth (Gibon et al., 2004; Baena-González and Sheen, 2008). Under such conditions, it may be more efficient to have a portion of proteins degraded and consumed as an energy source. Indeed, autophagy plays an important role during fluctuation in energy availability (Izumi et al., 2013). Several autophagy-deficient mutants show high sensitivity to extended darkness or nutrient starvation (Doelling et al., 2002; Thompson et al., 2005; Phillips et al., 2008; Suttangkakul et al., 2011). As a central growth regulator, BES1 is both transcriptionally and post-translationally regulated by sucrose under low-energy conditions (Zhang et al., 2015), and BES1 is targeted for autophagy-mediated degradation to slow down plant growth under fixed-carbon starvation (Nolan et al., 2017). Our results indicate that BAF1 is transcriptionally induced by sucrose starvation and ubiquitinates BES1. This in turn leads to BES1 degradation through selective autophagy to slow down plant growth, establishing a mechanism for plants to cope with this environmental/nutritional change.

SINAT2 E3 ligase was shown to interact with DSK2 and to be involved in targeting BES1 for autophagic degradation during starvation (Nolan et al., 2017). However, a recent study showed that SINAT family proteins also facilitate ATG13 ubiquitylation and stability to directly regulate both bulk and selective autophagy (Qi et al., 2020), which makes SINAT2’s role in BES1 selective autophagy more complex. The strong sucrose starvation phenotype of a SINAT2 mutant (Nolan et al., 2017) may include the effect from BES1 as well as other regulators affected by autophagy pathway. The relationship between BAF1 and SINATs in BES1 selective autophagy would be an interesting area for future exploration.

In this study, we examined the relationship between BAF1 and SINAT2 in terms of BR-regulated growth. We found that *baf1 SINAT RNAi* double mutant had much longer hypocotyls than single mutants with or without BRZ in the light. However, in the dark, *SINAT RNAi* was significantly shorter than WT, and *baf1SINAT RNAi* double mutant was like *baf1* single mutant. A previous study shows that SINATs control the light-mediated stability of BES1 (Yang et al., 2017) and SINATs likely have other substrates in the dark contributing to hypocotyl elongation. Together, BAF1 and SINATs appear to function redundantly in BR-mediated growth in the light (Supplemental Figure S6).

BAF1 is a novel F-box family E3 ubiquitin ligase with no homologs and without any functional annotation in TAIR (https://www.arabidopsis.org). As an F-box protein, BAF1 is predicted to be a component of an SCF ubiquitin–E3 ligase complex. F-box domains function to mediate the interaction of the F-box protein with SKP1 (Kipreos and Pagano, 2000). If the substrate recognition domain of an F-box protein is expressed without the F-box domain, it will function in a dominant-negative manner by competitively inhibiting ubiquitination of substrates by functional endogenous F-box proteins (Lee et al., 2018). Indeed, the BAF1 F-box decoy (BAF1-ΔF) strongly interacted with BES1 in both the cytoplasm and nucleus and stabilized both phosphorylated and dephosphorylated BES1 in vitro and in vivo. BAF1-ΔF has stronger phenotypes than *baf1* in terms of BES1 protein stability and hypocotyl elongation under some conditions (Figures 3, 4), suggesting that the dominant negative BAF1 affected other F-box proteins in degrading BES1. MAX2 is an F-box protein mediating BES1 degradation to regulate shoot branching through strigolactone signaling (Wang et al., 2013). We examined *baf1 max2* in terms of BR-regulated growth and found that *baf1 max2* double mutant had longer hypocotyls than single mutants in both dark and light conditions under BRZ treatment (Supplemental Figure S6). This indicates that BAF1 also functions redundantly with MAX2 under reduced BR conditions, although BAF1 and MAX2 share low similarity.

To date, very few F-box proteins have been defined to function in selective autophagy in Arabidopsis. One example of an F-box protein implicated in autophagy is the viral suppressor of RNA silencing P0 protein, which targets plant ARGONAUTE1 (AGO1) protein for degradation via an autophagy-related process (Baumberger et al., 2007; Michaeli et al., 2019). Our study identified an F-Box protein, BAF1, that functions to ubiquitinate and degrade BES1 through selective autophagy under sucrose starvation or reduced BR conditions. BAF1-mediated selective autophagy of BES1 is dependent on the receptor DSK2 (Figure 9, C). While loss-of-function of *BAF1* mutants had increased BES1 protein levels and BR-regulated growth, reduced BES1 selective autophagy, and were more sensitive to sucrose starvation, gain-of-function *BAF1* plants had opposite phenotypes. Although multiple E3 ubiquitin ligases have been identified to ubiquitinate and degrade BES1, none of the loss-of-function mutants for these genes, including *baf1*, *max2*, *cop1*, *SIANT RNAi*, *pub39/40/41*, display a constitutive BR phenotype as strong as *bes1-D* (Wang et al., 2013; Kim et al., 2014, 2019; Yang et al., 2017). *bes1-D* mutation in the PEST domain of BES1 leads to accumulation of BES1 by more than 20-fold and a constitutive BR response phenotype (Yin et al., 2002). Future studies should determine if some of these E3 ubiquitin ligase families function redundantly or complementarily to degrade BES1 under specific developmental or environmental conditions.

## Materials and methods

### Plant materials and growth conditions

*Arabidopsis thaliana* accession Columbia (Col-0) was used along with the previously described mutants: *atg5* (Thompson et al., 2005), *DSK2 RNAi* (Lin et al., 2011; Nolan et al., 2017), *SINAT RNAi* (Yang et al., 2017), *max2* (Wang et al., 2013), and *bes1-D* mutant (Yin et al., 2002) integrated into Col-0 background (Vilarrasa-Blasi et al., 2014). T-DNA insertion mutant *baf1* (GABI_001F08) was obtained from GABI-Kat database (https://www.gabi-kat.de) and confirmed before use. The *35S:BES1-GFP* seeds are kindly provided by Dr. Ana I. Caño-Delgado (CSIC-IRTA-UAB). Homozygous progeny of crosses *baf1 SIANT RNAi*, *baf1 max2*, and *BAF1 OX14 atg5* were used, while F2 and F3 progeny of cross *BAF1 OX14 DSK2 RNAi* were used in this study. Seeds were sterilized with 70% (v/v) ethanol and 0.1% (v/v) Triton X-100 for 15 min, followed by two washes with absolute ethanol and drying on filter paper. Sterilized seeds were grown on 1/2 Linsmaier and Skoog (LS, LSP03-1LT, Caisson Laboratories) plus 1% sucrose plates, or seedlings later were transferred into soil (SS#1-F1P, SunGro) at 22 °C under long-day (16-h light/8-h dark) conditions with a photon fluence rate of ∼100–120 µmol m^−2^ s^−1^. For relatively weak light, the photon fluence rate is ∼60 µmol m^−2^ s^−1^.

### Plasmid constructs and generation of transgenic plants

Plasmid constructs were generated either via restriction enzyme or Gateway technology (Invitrogen) and were confirmed by DNA sequencing. All the primers with restriction sites used in this study are listed in Supplemental Table S1. All the constructs used in this study are listed in Supplemental Table S2. Plasmid constructs were transferred into *Agrobacterium tumefaciens* strain GV3101 and used to transform plants by the floral-dip method (Clough and Bent, 1998). *35S:BAF1-FLAG* and *35S:BAF1-ΔF-FLAG* overexpressing plants were screened on 1/2 LS plates supplemented with 50 mg/L kanamycin and further confirmed by immunoblotting using anti-FLAG (F7425, Sigma–Aldrich) antibodies. *35S:BAF1-MYC* and *35S:BAF1-ΔF-MYC* overexpressing plants in the background of *bes1-D* or *35S:BES1-GFP* were screened on 1/2 LS plates supplemented with 75 mg/L gentamycin and further confirmed by immunoblotting using anti-c-MYC (C3956, Sigma–Aldrich) antibodies. Homozygous T3 lines of these transgenics were further identified for experimental use in this study. *35S:BAF1-FLAG* overexpressing plants in *DSK2 RNAi* background were screened on 1/2 LS plates supplemented with 50 mg/L kanamycin plus 10 mg/L herbicide Basta and T2 and T3 lines were used.

### BRZ and BL response assays

For BRZ response assays in the dark, sterilized seeds were grown on 1/2 LS medium with 1% sucrose containing DMSO (control, BRZ0) or indicated concentrations of BRZ (BRZ100, 250 and 500 nM) (Asami et al., 2000). Plates were stored in the dark at 4 °C for 4 days, then exposed to light for 6–8 h, and then kept in darkness for 7 days. Seedlings were imaged and hypocotyl lengths were measured using ImageJ software (https://imagej.nih.gov/ij/). For BRZ response assays in the light, plates were kept under continuous light for 10 days at 22 °C, and the longest petiole of each plant was measured by a ruler. For BRZ response assays in the relatively weak light, plates were kept under continuous light for 10 days at 22 °C, and the hypocotyl of each plant was measured by a ruler.

BL response assays were conducted similarly to the BRZ experiments described above with the exception that the plants were grown for 7 days under continuous light at 22 °C (Li et al., 2010). Control (DMSO solvent only, BL0) and different BL treatments (5, 10, 20, 50, and 100 nM) were tested. Approximately 10 seeds for each genotype were grown on one plate, and there were four individual plates per BRZ or BL concentration.

### Long-term sucrose starvation assay

Seedlings were grown on 1/2 LS plates without sucrose in the light for 4 days and then transferred to darkness for 9–14 days as indicated. For each indicated time, plates were put back into the light for a 7-day-recovery period to assess survival. Plants with new growth were considered as surviving (Wang et al., 2020). For each replicate, 30 seedlings were used for each genotype per treatment. Data were derived from six to nine biological replicates.

### Yeast two-hybrid assay

The GAL4 DNA binding domain (BD) of pGBKT7 (Clontech) was fused to the N-terminus of full-length BES1 or BES1-D protein, and the GAL4 activation domain (AD) of pGADT7 (Clontech) was fused to the N-terminus of BAF1 protein. Two constructs were co-transformed into yeast strain Y187 using the LiAc method and screened using SD-Trp–Leu medium. The optical density (OD) was read as absorbance at 600 nm using a multi-mode plate reader (Eppendorf AF2200). β-Galactosidase activity was assessed using a commercially available luminescent β-galactosidase substrate Beta-Glo (Promega, E4740), which is cleaved to release d-luciferin as a firefly luciferase substrate (de Almeida et al., 2008). The LUC values were normalized to the culture absorbance OD600.

### BiFC assays

BiFC assays were conducted as previously described (Ye et al., 2012). BES1, BAF1, and BAF1-ΔF were cloned upstream of either the N- or C-terminus of YFP and introduced into *A.* *tumefaciens* (strain GV3101). MYC2-nYFP was introduced as a negative control of BES1-nYFP. Transformed *Agrobacterium* colonies were cultured in LB medium containing 0.2 mM acetosyringone for 1–2 days. Collected cells were washed and resuspended to OD_600_ of 0.9 with infiltration buffer (10 mM MgCl_2_, 10 mM MES, pH 5.7, 0.2 mM acetosyringone). *Agrobacterium* carrying nYFP and cYFP constructs were mixed equally and infiltrated into the lower surface of *N. benthamiana* leaves (Yu et al., 2008). After 36 h, YFP signals were detected using a Leica SP5 × MP confocal microscope equipped with an HCS PL APO CS 20 × 0.7 oil objective. Images were processed with LAS AF software (Leica Microsystems).

### In vitro pull-down assays

GST pull-down assays were conducted using GST or MBP fusion proteins. BES1 was cloned into pET-MBP-H vector (Nolan et al., 2017), while BAF1 and BAF1-ΔF were cloned into pET42a (Novagen). Recombinant proteins were produced in *Escherichia* *coli* strain BL21, which were induced by 300 µM isopropylthio-β-galactoside (IPTG) for 24 h at 16 °C and purified using either amylose resin (NEB) or glutathione beads (Sigma–Aldrich). GST or each GST-tagged fusion were mixed with the MBP fusion protein in 1 mL GST-pull-down buffer (50 mM Tris–HCl, pH 7.5, 200 mM NaCl, 0.5% Triton X-100, and 0.5 mM β-mercaptoethanol, proteinase inhibitor cocktail) and incubated at room temperature for 2 h on a tube rotator. Twenty microliters of GST beads was used per reaction and the beads were pre-blocked overnight with 1 mg/mL bovine serum albumin (BSA) and BL21 extract at 4 °C to reduce background. Pre-blocked beads were added to the reaction and incubation continued for an additional 30 min. GST beads were washed in GST-pull-down buffer seven to eight times and then eluted in 2× SDS sample buffer (100 mM Tris–HCl pH 6.8, 4% (w/v) SDS, 20% (v/v) glycerol, 0.2% (w/v) bromophenol blue, 0.2 M β-mercaptoethanol). The pull-down protein was separated on an SDS–PAGE gel and detected with mouse anti-MBP antibody (E8032S, New England Biolabs). GST-pull-down experiments were repeated two to three times with similar results.

### Co-immunoprecipitation

Co-IP experiments were conducted as previously described (Xie et al., 2019). *Arabidopsis* protoplasts co-transformed with tested GFP and FLAG constructs (*35S:BAF1-FLAG*, *35S:BAF1-ΔF-FLAG*, and *35S:BES1-GFP*) were used in this study. After overnight culture, transformed protoplasts were harvested and homogenized in Co-IP buffer (50 mM Tris–HCl, pH 7.5, 150 mM NaCl, 10% (v/v) glycerol, 0.1% (v/v) Nonidet P-40, 1 mM phenylmethysulfonyl fluoride, 20 µM MG132, and proteinase inhibitor cocktail) for 1 h at 4 °C with rotation. 5 µg FLAG M2 antibody (F1804, Sigma–Aldrich) was pre-bound to 40 µL protein G Dynabeads (10003D, Thermo Fisher Scientific) for 30 min in 1× phosphate-buffered saline (PBS) buffer with 0.02% Tween 20 at room temperature. The beads were washed once with the same PBS buffer and resuspended in Co-IP buffer. After protein extraction, 10 µL of anti-FLAG pre-bound Dynabeads was added to each sample for another 1.5 h incubation at 4 °C with rotation. Dynabeads was precipitated using DynaMagnetic rack (12321D, Thermo Fisher Scientific) and washed twice with Co-IP buffer w/Nonidet P-40 and three times with Co-IP buffer w/o Nonidet P-40. The IP products were eluted in 2× SDS sample buffer and used for immunoblotting with rabbit anti-GFP (A11122, Invitrogen) and rabbit anti-FLAG antibody (F7425, Sigma–Aldrich) at 1:1,000 dilution. Co-IP experiments were repeated two to three times with similar results.

### In vivo ubiquitination assays

Harvested *N. benthamiana* or Arabidopsis tissues were ground to a fine powder in liquid nitrogen and extracted in IP buffer (50 mM Tris–HCl, pH 7.5, 150 mM NaCl, 10% (v/v) glycerol, 0.5% (v/v) Nonidet P-40, 1 mM phenylmethysulfonyl fluoride, 20 µM MG132, and proteinase inhibitor cocktail). Extract was clarified using Miracloth (475855, EMD Millipore) for filter and by two rounds of centrifugation at 13,000 *g* for 10 min at 4 °C, and the supernatant was incubated with FLAG M2 antibody pre-bound protein G Dynabeads or BES1 antibody pre-bound protein A Dynabeads for 2 h with gentle rocking at 4 °C. Beads were washed twice with IP buffer containing 0.5% Nonidet P-40 and twice with IP buffer without Nonidet P-40. The IP product was resuspended in 2× SDS sample buffer and resolved on 8% SDS–PAGE gels against anti-UBQ (3933S, Cell Signaling Technology), anti-K63-UBQ (5621S, Cell Signaling Technology), and anti-K48-UBQ (4289S, Cell Signaling Technology) antibodies.

### Immunoblotting

For IP samples, 1 g of each sample was ground in liquid nitrogen and extracted with 2.5 mL IP buffer; for regular total protein extraction from Arabidopsis, 50 mg tissues were collected and flash frozen in liquid nitrogen and ground directly in 200 µL 2× SDS sample buffer (usually 3–4 volumes by fresh weight). For regular total protein extraction from *N. benthamiana*, five leaf discs (7 mm in diameter) were collected from each sample and flash frozen in liquid nitrogen and ground directly in 150 µL 2× SDS sample buffer. For immunoblotting, samples were boiled for 5 min and chilled on ice for 1–2 min before centrifugation at 13,000 *g* for 5 min at room temperature. A total of 10–20 µL samples were loaded in 8% or 10% SDS–PAGE gels. After protein separation by electrophoresis, gels were transferred to nitrocellulose membranes (1620115, BioRad) using Trans-Blot Turbo Transfer System (BioRad). Membranes were blocked in 5% non-fat milk in Tris-buffered saline with Tween 20 (TBST, 20 mM Tris, pH 8.0, 150 mM NaCl, 0.1% Tween 20) at room temperature for 1 h and then incubate overnight with the primary antibody at 4 °C. The following antibodies were used in this study in conjunction with appropriate secondary antibody-HRP conjugates: anti-BES1 (Yu et al., 2011), anti-GFP, anti-FLAG, anti-Ubiquitin (PratelLi et al., 2012), anti-MBP, anti-MYC (C3956, Sigma–Aldrich), anti-IWS1, anti-UBQ, anti-K48-UBQ, and anti-K63-UBQ. The information on antibodies used in this study is provided in Supplemental Table S3. Quantified relative band intensity was analyzed using Image J.

### Protein co-expression in *N. benthamiana* and inhibitor treatments

*Agrobacterium* carrying BES1 and BAF1-FLAG or BAF1-ΔF-FLAG constructs were mixed with p19 and infiltrated in *N. benthamiana* leaves as described for BiFC assays. Leaf discs were typically collected 2 days post-infiltration for protein extraction. For experiments using inhibitors, 12 h post-infiltration, infiltration medium containing DMSO, 50 µM MG132 (M7449, Sigma–Aldrich), 40 µM E64d (E8640, Sigma–Aldrich), 1 µM ConA (C9705, Sigma–Aldrich), or 2 µM BafA1 (B1793, Sigma–Aldrich) was infiltrated into the same leaf area as previously infiltrated. Samples were collected 24 h after addition of inhibitors. All combinations were performed in the same leaf to ensure any differences are not due to leaf variation or inhibitor ineffectiveness.

### Transient expression assays and autophagy detection in Arabidopsis protoplasts

Leaves from 4-week-old Arabidopsis plants grown under long-day conditions were collected for protoplast isolation. The “Tape-Arabidopsis Sandwich” technique was employed to peel leaves (Wu et al., 2009) and procedures followed a previously described protocol (Yoo et al., 2007). Protoplasts were resuspended to final concentration of 3.0–3.5 × 10^5^/mL in MMg solution (400 mM mannitol, 15 mM MgCl_2_, and 4 mM MES pH5.7). Plasmid DNA was prepared using Maxiprep kits (NA0310, Sigma–Aldrich) and set to at 1 µg/µL final concentration. Transformations were carried out in 2 mL round bottom centrifuge tubes. Twenty micrograms of each construct was introduced into 300 µL protoplasts by adding 340 µL of PEG solution (40% PEG4000, 200 mM mannitol, and 100 mM CaCl_2_). After transformation, protoplasts were washed and incubated in 2 mL of W5 solution (154 mM NaCl, 125 mM CaCl_2_, 5 mM KCl, 2 mM MES, pH 5.7) overnight.

For sucrose starvation treatment, protoplasts were incubated in W5 solution without sucrose or with 0.5% (w/v) sucrose as control at RT for 36 h. Protoplasts were observed by epifluorescence microscopy (Carl Zeiss Axio Imager.A2, Germany). For observation of BES1-GFP and mCherry-ATG8e labeled autophagosomes, FITC and TRITC filters were used, respectively. Protoplasts with more than three visible autophagosomes were counted as active for autophagy (Wang et al., 2020). A total of 100 protoplasts were observed per treatment per genotype, and the percentage of protoplasts with active autophagy was calculated and averaged from three independent experimental replicates. For colocalization of BES1-GFP and mCherry-ATG8e in protoplasts, both constructs were co-transformed into protoplasts which were subjected to sucrose starvation for 36 h. Confocal microscopy was performed with Zeiss Laser Scanning Microscope 700 (LSM700). GFP and mCherry fluorescent signals were excited with 488 and 555 nm, respectively. The signals were then collected using the emission filters of 555 nm for GFP and 640 nm for mCherry.

### Detection of autophagosomes in Arabidopsis roots

For GFP-ATG8e labeled autophagosomes, GFP-ATG8e was transformed into *baf1*, *BAF1 OX14*, and *BAF1-ΔF OX46* backgrounds and were screened on 1/2 LS plates supplemented with 20 mg/L hygromycin, and three individual T2 lines from each background were used. For sucrose starvation, 7-day-old seedlings were transferred to 1/2 LS media with or without sucrose for an additional 3 days in the dark. For nitrogen starvation, 7-day-old seedlings were transferred to 1/2 LS media with or without nitrogen for an additional 3 days in the light. After treatments, the GFP-ATG8e labeled autophagosomes in the roots were observed by epifluorescence microscopy (Carl Zeiss Axio Imager.A2) using a GFP filter. Two to three representative images in the root elongation zone were photographed per seedling and the number of GFP-ATG8e puncta in each image was counted and averaged. A total of ten seedlings were observed per treatment. Representative confocal images were taken in the roots by Zeiss Laser Scanning Microscope 700 (LSM700) with a 63× oil immersion objective. GFP fluorescent signals were collected with excitation and emission at 488 and 555 nm, respectively.

For BES1-GFP labeled autophagosomes, 7-day-old seedlings were transferred to 1/2 LS liquid media with or without sucrose or nitrogen for 16 h and roots were observed by Zeiss Laser Scanning Microscope 700 (LSM700) with a 63× oil immersion objective. GFP fluorescent signals were collected with excitation and emission at 488 and 555 nm, respectively. Three to five representative images in the root elongation zone were photographed per seedling and the number of BES-GFP puncta in each image was counted and averaged. A total of five seedlings was observed per treatment.

### In vivo degradation assays

For in vivo BES1 degradation assay, 7-day-old seedlings of different genotypes (WT, *baf1*, *35S:BAF1-FLAG OX14* and *OX16* lines, and *35S:BAF1-ΔF-FLAG OX18* and *OX46* lines) grown on 1/2 LS control with or without 2 µM BRZ were treated with 1 mM CHX in 1/2 LS liquid medium for 0, 1, 2, 4, and 6 h. For samples with inhibitors, 7-day-old seedlings of WT and *35S:BAF1-FLAG OX14* grown on 1/2 LS treated with 1 mM CHX with or without 40 µM E64d or 50 µM MG132 in 1/2 LS liquid medium for 0, 1, 2, and 4 h. Total proteins were extracted in 2× SDS sample buffer and samples were immunoblotted using anti-BES1 antibody. The band intensity was quantified using ImageJ software (Schindelin et al., 2012). Experiments were conducted two to three times with similar results.

### QuantSeq analysis

Total RNA was extracted from 7-day-old seedlings growing on control 1/2 LS media or plus 250 nM BRZ media using Zymo DirecZol Kit (Zymo Research), and genomic DNA contamination was removed using RNase-free DNase in column during RNA extraction according to the manufacturer’s protocols. Library was constructed using QuantSeq 3′ mRNA-Seq Library Prep Kit from Illumina. Sequencing was performed on a HiSeq 4000 system with 50-bp single end reads. FASTQ files for each sample were subject to quality control and trimming and mapped to The Arabidopsis Information Resource 10 (TAIR10) genome using the BlueBee Arabidopsis (TAIR10) Lexogen QuantSeq 2.2.2 FWD pipeline. Principal component analysis was used to examine the data. All raw and processed Quantseq data described in this study have been deposited in the Gene Expression Omnibus (https://www.ncbi.nlm.nih.gov/geo/query/acc.cgi?acc=GSE155219). For differential expression analysis, statistics was performed using the PoissonSeq package in R (Li et al., 2012). For comparisons of DEGs, Venn diagrams were generated using Venny 2.1.0 (https://bioinfogp.cnb.csic.es/tools/venny/). Over-enrichment *P*-value was calculated based on hypergeometric distribution (https://systems.crump.ucla.edu/hypergeometric/index.php). Clustering was performed using the “heatmap.2” function of the gplots package in R (https://cran.r-project.org/web/packages/gplots/index.html). The average normalized expression of all samples was computed and set as reference, all samples’ normalized expression was compared with the average expression and the color was presented in log scale [log2(sample_expression/average_expression)].

### RT-qPCR

Total RNA was isolated from 7-day-old seedlings using a RNeasy Plant Mini Kit (74904, QIAGEN) and genomic DNA was removed using RNase-free DNase Set (79254, QIAGEN) in column during RNA extraction. The first strand cDNA was synthesized with an iScript cDNA Synthesis Kit (1708891, BioRad). Real-time PCR was performed using SYBR Green PCR Master Mix (4309155, Applied Biosystems) on the StepOnePlus Real-Time PCR System (Applied Biosystems) with 40 cycles. The relative gene expression was determined by applying the 2^−^^ΔΔCT^ (CT: cycle threshold) method and normalized to the expression of the reference gene *ACTIN2* (*AT3G18780*). RT-qPCR was performed on three technical replicates of two to three independent biological RNA samples. Primers used for qPCR are provided in Supplemental Table S1 and melt curves were performed on each primer pairs to confirm gene–product specificity.

### Statistical analysis

SPSS 27.0 software (IBM) was used for statistical data analysis. The data were expressed as the mean ± standard error of the mean (SEM) and were subjected to one-way analysis of variance (ANOVA) Tukey’s multiple range tests (*P *<* *0.05). ANOVA data are provided in Supplemental Data Set S2.

## Accession numbers

QuantSeq data from this article have been deposited in the Gene Expression Omnibus (GSE155219). The accession numbers of the main genes discussed in this article are: *BAF1* (*AT1G76920*), *BES1* (*AT1G19350*), *DSK2A* (*AT2G17190*), *ATG5* (*AT5G17290*), *SINAT2* (*AT3G58040*), and *MAX2* (*AT2G42620*).

## Supplemental data

**Supplemental Figure S1.** BAF1 and BES1 interaction revealed by GST pull-down and Co-IP assays.

**Supplemental Figure S2.** A set of BiFC negative controls using BAF1 and MYC2 in *N. benthamiana*.

**Supplemental Figure S3.** BAF1 still mediates BES1-D degradation and generated ubiquitination of BES1 in vivo.

**Supplemental Figure S4.** Protein stability assay of IWS1 protein serving as a negative control.

**Supplemental Figure S5.** BRZ response assay in the dark of two more *BAF1 OX* lines.

**Supplemental Figure S6.** BAF1 functions redundantly with other BES1 E3 ligases SINATs and MAX2.

**Supplemental Figure S7.** The response of BAF1-mediated BES1 degradation to inhibitors in Arabidopsis seedlings and *N. benthamiana*.

**Supplemental Figure S8.** The co-localization of BES1-GFP and mCherry-ATG8e in protoplasts from different genotypes.

**Supplemental Figure S9.** BAF1 mediates BES1 degradation largely through autophagy.

**Supplemental Figure S10.** BAF1 mediates plant sensitivity to long-term sucrose starvation.

**Supplemental Table S1.** Primer sequences used in this study.

**Supplemental Table S2.** Information on constructs used in this study.

**Supplemental Table S3.** Information on antibodies used in this study.

**Supplemental Data Set S1.** Genes regulated by BAF1, BES1-D, and BRZ.

**Supplemental Data Set S2.** ANOVA results in this study.

## Supplementary Material

koab210_Supplementary_Data

## Notes

P.W., T.M.N., and Y.Y. designed the project. P.W. performed most of the experiments with the following exceptions: T.M.N. made some of the constructs and performed ubiquitination assay and BiFC in *N. benthamiana*; P.W. and T.M.N. conducted QuantSeq; N.M.C. analyzed QuantSeq data; H.J. generated the heatmap; C.M.-S. assisted with ubiquitination assays; H.G., D.C.B., and J.W.W. provided critical guidance and data analysis on experiments and project; P.W. and Y.Y. wrote the manuscript with inputs from T.M.N., H.G., D.C.B., and J.W.W.

The author responsible for distribution of materials integral to the findings presented in this article in accordance with the policy described in the Instructions for Authors (https://academic.oup.com/plcell) is Yanhai Yin (yin@iastate.edu).
