# Supplementary material for: The F-box E3 ubiquitin ligase BAF1 mediates the degradation of the brassinosteroid-activated transcription factor BES1 through selective autophagy in Arabidopsis
Source: Plant Cell. 2021 Aug 26;33(11):3532–54. doi: 10.1093/plcell/koab210 (PMC8566207; doi:10.1093/plcell/koab210)
Supplement: koab210_Supplementary_Data [file koab210_supplementary_data.zip › tpc.21.00451_SupplementalFiguresandTables.pdf]

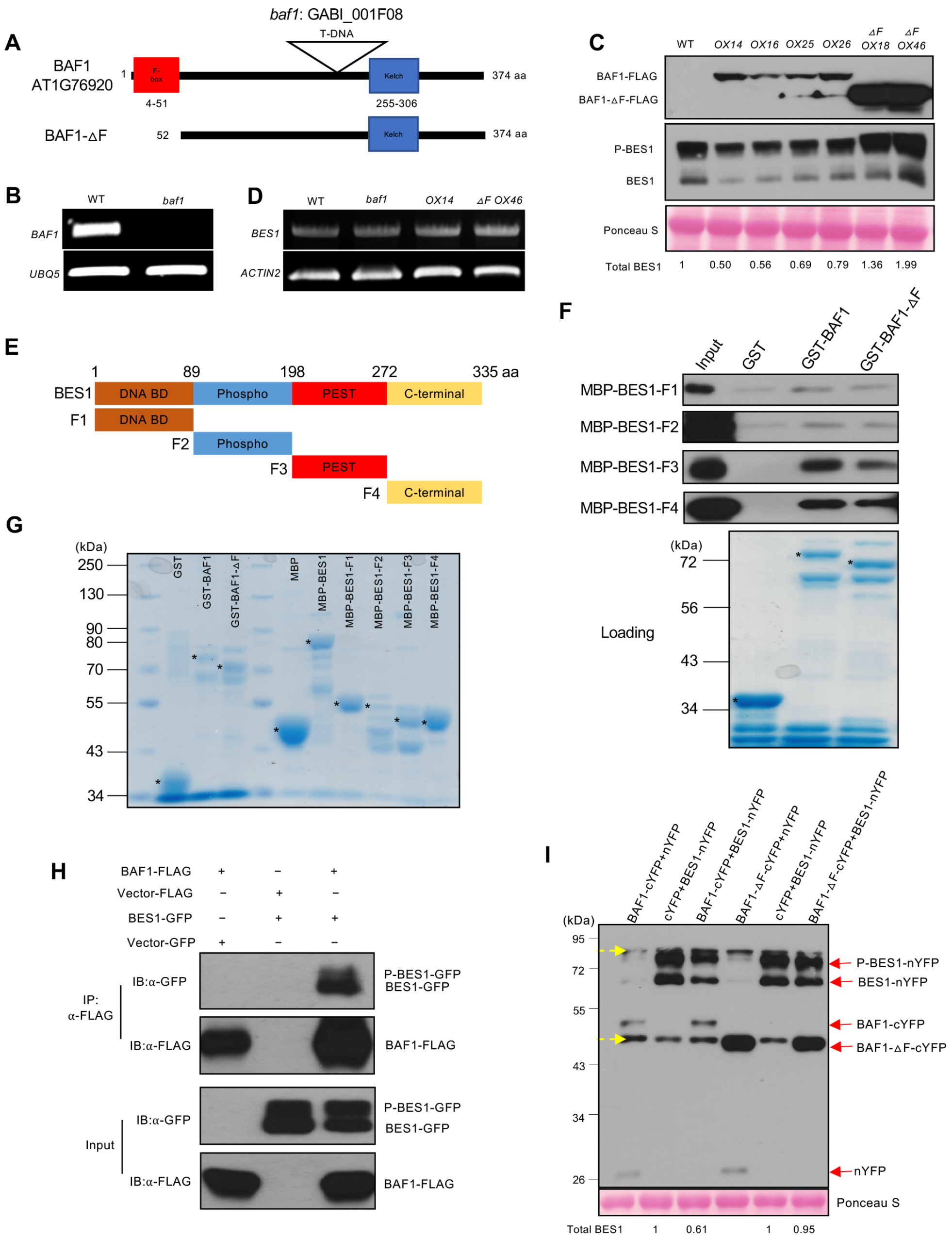

Supplemental Figure S1. BAF1 and BES1 interaction revealed by GST pull-down and Co-IP assays. (Supports Figure 1). (A) Schematic diagram of BAF1 protein information with domains and T-DNA insertion location. (B) RT-PCR indicated that expression of *BAF1* was knocked out in *baf1* mutant. *UBQ5* serves as internal control. (C) Protein expression in *BAF1-FLAG OX* lines and *BAF1-ΔF-FLAG OX* lines. 7-d-old seedlings were collected and analyzed by immunoblotting with anti-FLAG (rabbit) and anti-BES1 antibodies. (D) RT-PCR indicated that expression of *BES1* was not affected in *baf1* mutant and *BAF1* transgenic lines. *ACTIN2* serves as internal control. (E) Schematic diagram of BES1 protein fragments with different domains (DNA-binding domain, Phosphorylation domain, PEST domain and C-terminal domain) used for following GST pull-down assay. (F) The interaction of BAF1 with different BES1 domains in GST pull-down assays. The loading of GST, GST-BAF1 and GST-BAF1-ΔF proteins are shown by a Coomassie-stained gel (bottom). Asterisks indicated the desired protein. MBP-BES1 fragments were detected by immunoblotting with anti-MBP (mouse) antibody. (G) A full gel of purified proteins used for GST pull-down assay. Asterisks indicated the desired protein. (H) Another set of Co-IP assay showed BAF1 and BES1 interaction in Arabidopsis protoplasts. BAF1-FLAG and BES1-GFP as well as control vectors were co-transformed into Arabidopsis protoplasts overnight. Protein was immunoprecipitated with anti-FLAG (mouse) and detected with anti-FLAG (rabbit) and anti-GFP (rabbit) antibodies. (I) The protein expression of BiFC combinations. Samples were analyzed by immunoblotting with anti-GFP (rabbit, lab-made) antibody. Red arrows indicated the target protein expression while yellow arrows indicated unspecific band of the antibody. Ponceau S serves as loading control. Quantified relative band intensity of total BES1 protein was listed below using Image J

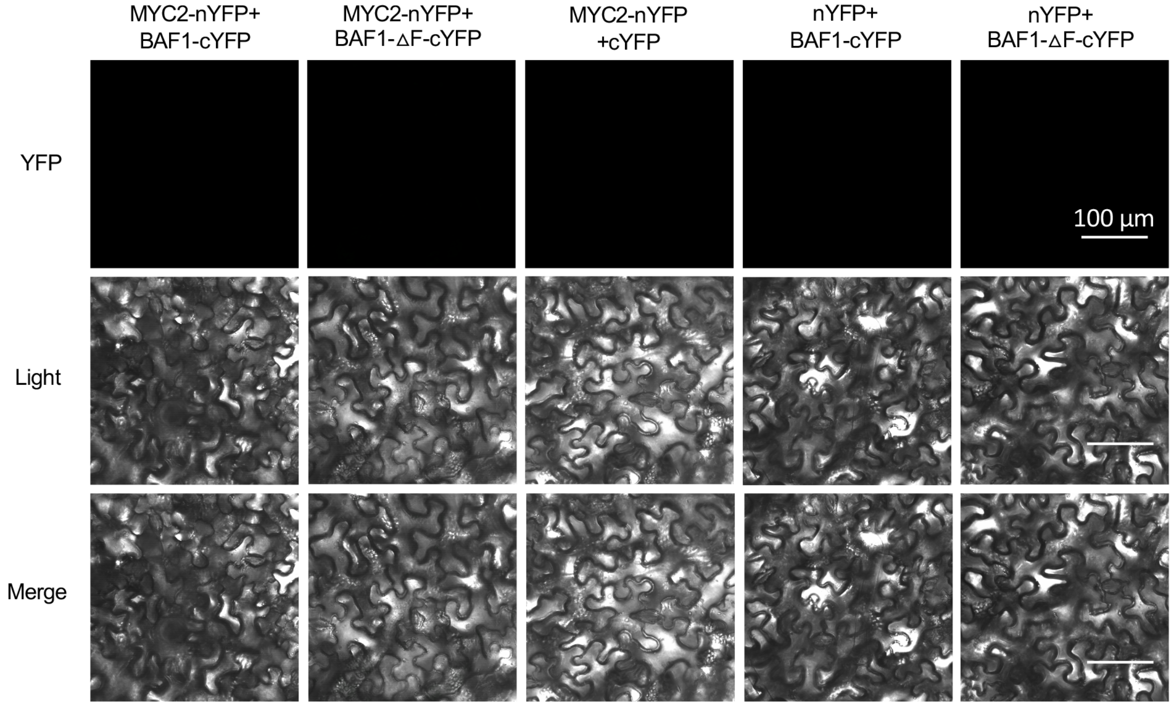

Supplemental Figure S2. A set of BiFC negative controls using BAF1 and MYC2 in *N. benthamiana*. (Supports Figure 1). Fluorescence, light or merged images of leaf cells are shown. Scale bar represents 100 µm.

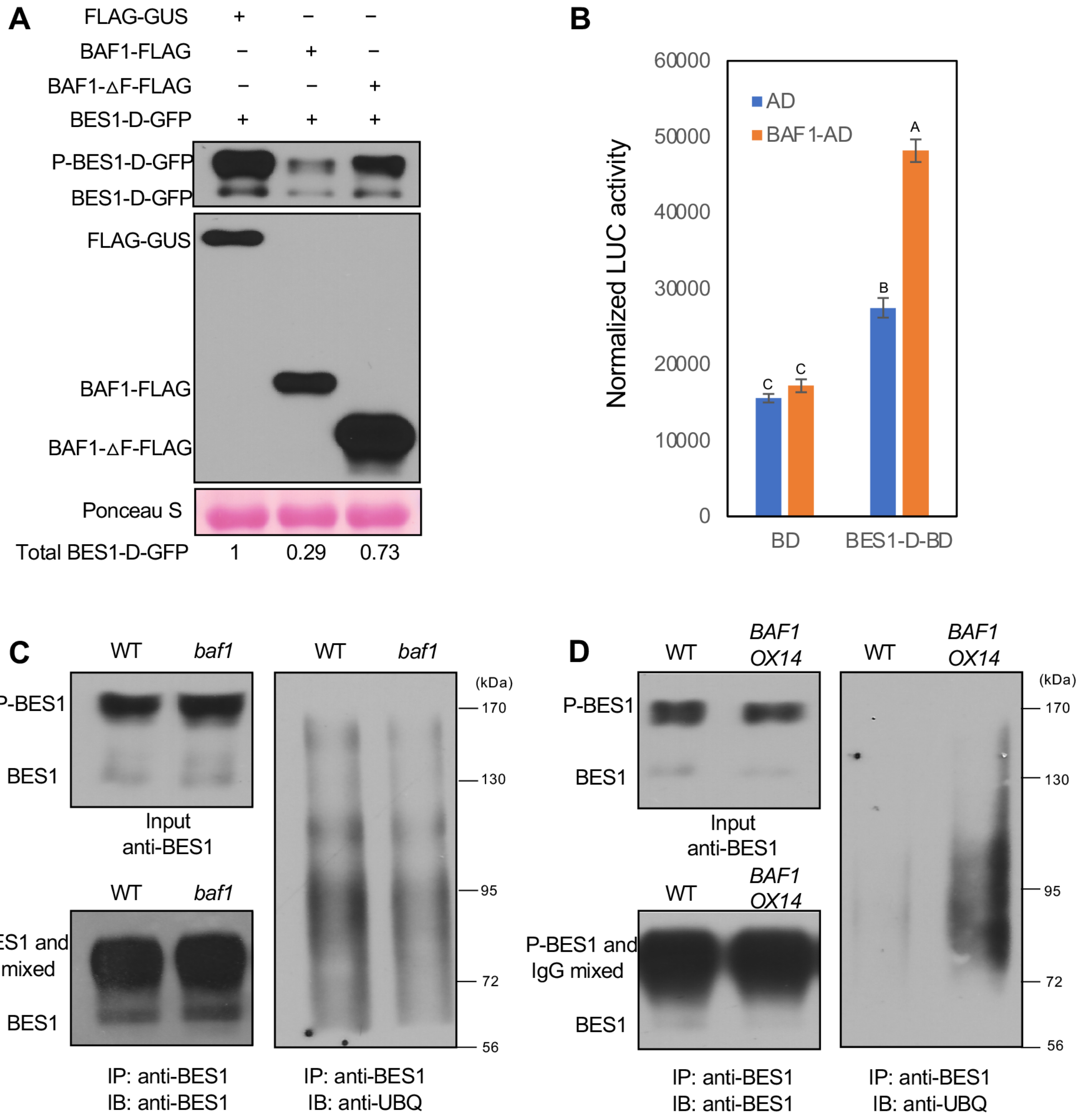

Supplemental Figure S3. BAF1 still mediates BES1-D degradation and generated ubiquitination of BES1 *in vivo*. (Supports Figure 2). (A) BAF1-FLAG, BAF1-ΔF-FLAG and BES1-D-GFP as well as control vectors were co-infiltrated into *N. benthamiana* for 2 d. Proteins were detected by immunoblotting with anti-GFP (rabbit) and anti-FLAG (rabbit) antibodies. Ponceau S serves as loading control. Quantified relative band intensity of total BES1-D-GFP protein was listed below using Image J. (B) BAF1 interacted with BES1-D in yeast as detected by β-galactosidase activity which was assessed using a commercial luminescent β-galactosidase substrate Beta-Glo. The GAL4 DNA binding domain (BD) of pGBKT7 was fused to the N-terminus of full-length BES1-D protein, and the GAL4 activation domain (AD) of pGADT7 was fused to the N-terminus of full-length BAF1 protein. Data represent mean ± SEM, n = 8. Different letters indicate significant difference according to one-way ANOVA Tukey's multiple range tests ($p < 0.05$). (C) and (D) *In vivo* ubiquitination of BES1 from WT, *baf1* and *BAF1 OX14* seedlings. BES1 protein was immunoprecipitated from 7-day-old seedlings by BES1 antibody. The immunoprecipitation product was analyzed by immunoblotting with anti-BES1 and anti-UBQ (rabbit) antibodies. The size of P-BES1 is close to that of rabbit IgG protein, so when detecting the immunoprecipitation product by anti-BES1 antibody, P-BES and IgG co-migrate, but the BES1 band can be seen clearly, indicating the successful immunoprecipitation.

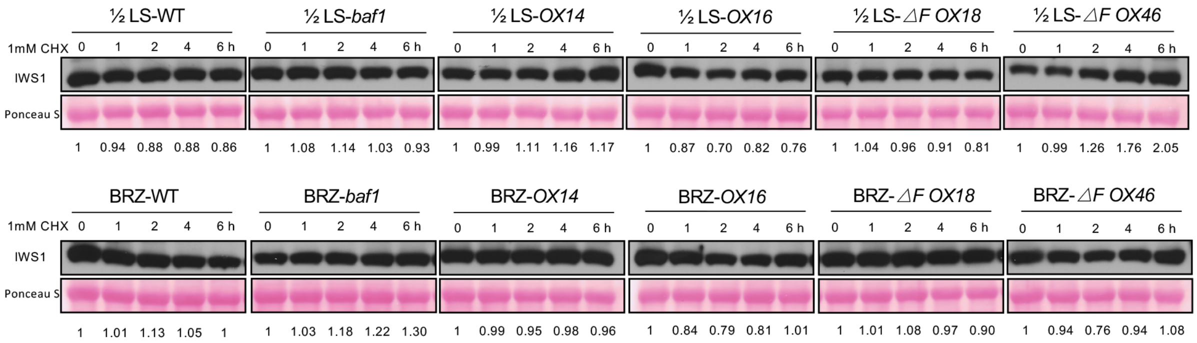

Supplemental Figure S4. Protein stability assay of IWS1 protein serving as a negative control. (Supports Figure 3). IWS1 protein stability was assessed by treating Arabidopsis seedlings grown on ½ LS media or ½ LS plus 2 μM BRZ with 1 mM CHX for the indicated times. WT, *baf1*, *BAF1-FLAG OX* lines and *BAF1-ΔF-FLAG OX* lines were examined. Samples were analyzed by immunoblotting with anti-IWS1(rabbit) antibody, and relative intensity of protein bands were quantified by ImageJ as shown below each gel. Ponceau S staining serves as loading control.

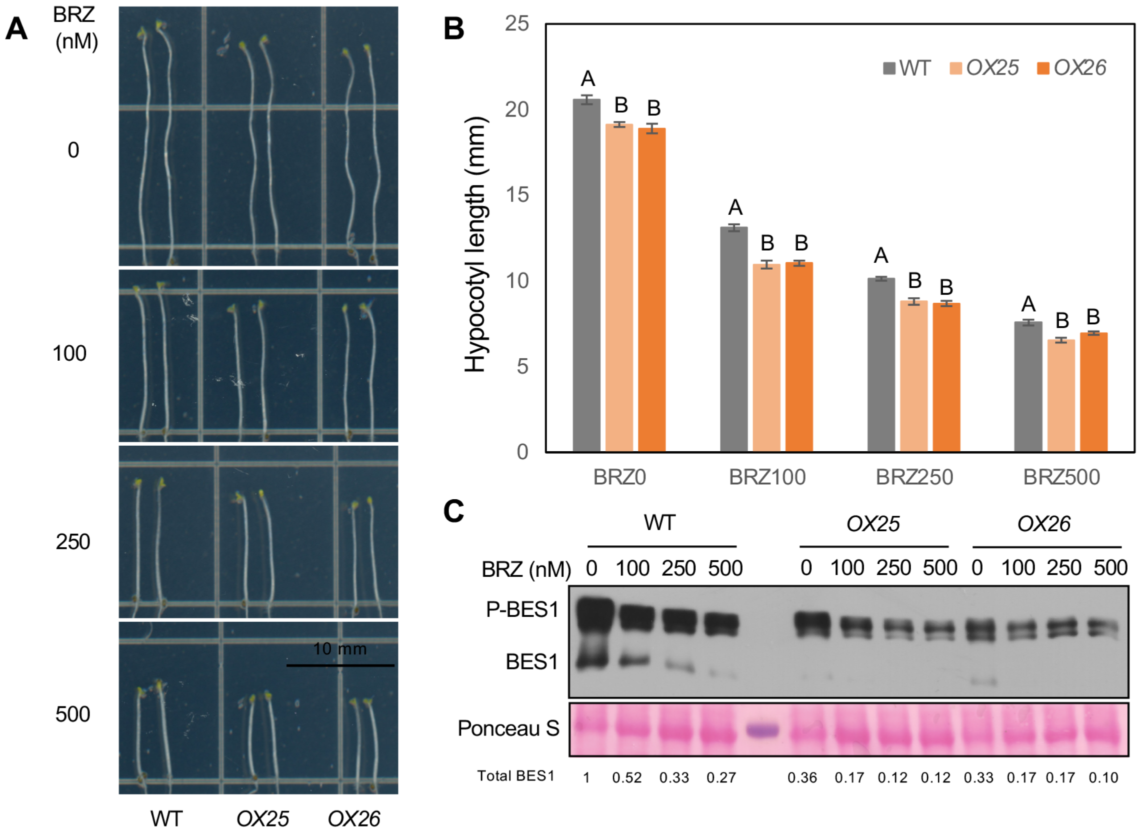

Supplemental Figure S5. BRZ response assay in the dark of two more *BAF1 OX* lines. (Supports Figure 4). WT, *BAF1-FLAG OX25* and *OX26* were examined. Seedlings were grown on ½ LS medium with different concentrations of BRZ (0, 100, 250 and 500 nM) for 7 days in dark (A). Hypocotyls were measured using ImageJ (B). Data represent mean ± SEM of 18 seedlings from three biological replicates (n = 18). Only significant differences between genotypes within one BRZ treatment were compared, which were indicated by different letters according to one-way ANOVA Tukey's multiple range tests (p < 0.05). Scale bar represents 10 mm. (C) BES1 protein level in the samples of (A). Whole seedlings were collected at the end of treatments and analyzed by immunoblotting with anti-BES1 antibody. BES1 and P-BES1 indicates unphosphorylated and phosphorylated BES1, respectively. Ponceau S staining serves as loading control. Quantified relative band intensity of total BES1 protein was listed below using Image J.

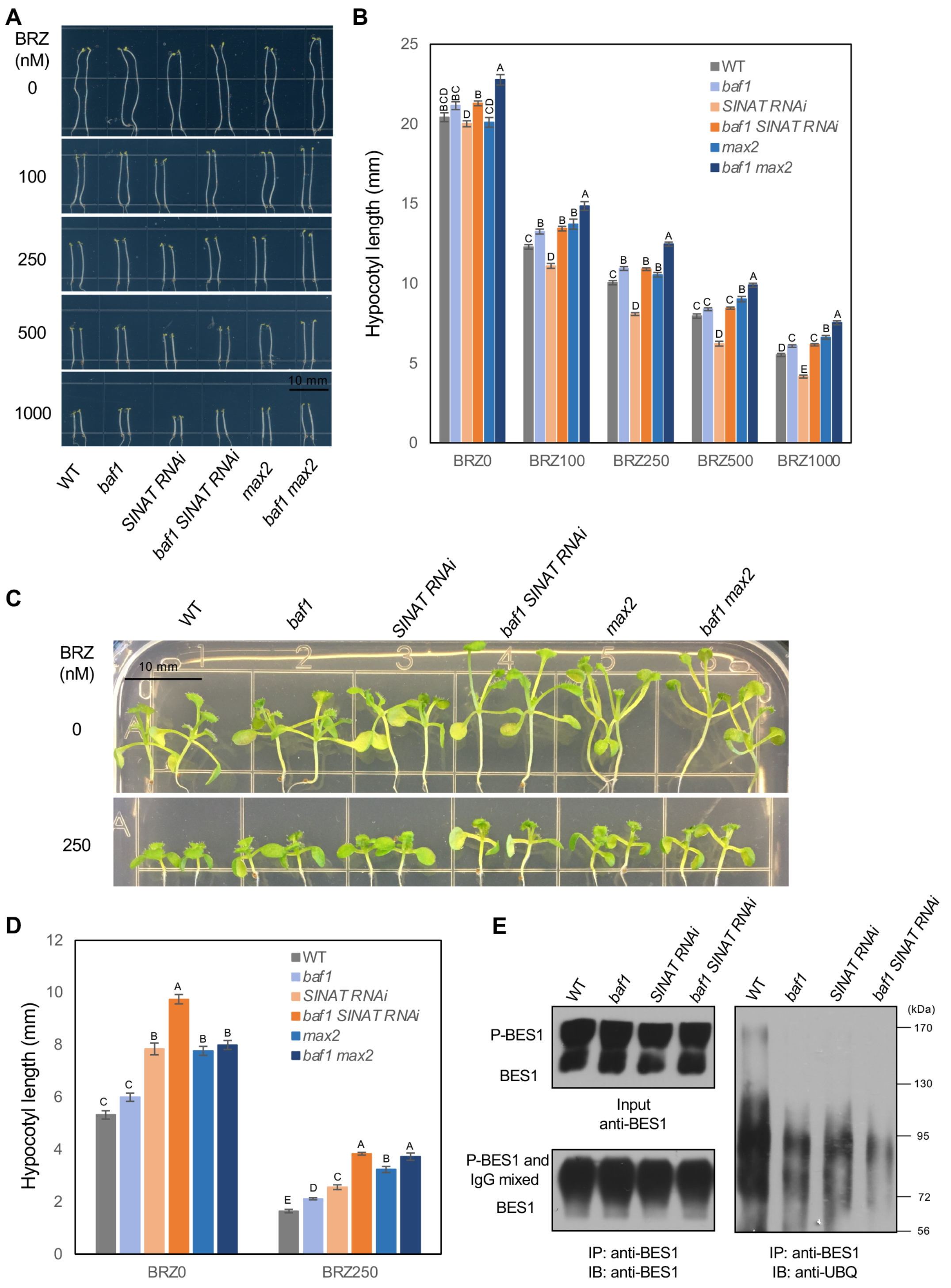

Supplemental Figure S6. BAF1 functions redundantly with other BES1 E3 ligases SINATs and MAX2. (Supports Figure 5). (A) and (B) BRZ sensitivity assay in the dark. WT, *baf1, SINAT RNAi, baf1SINAT RNAi, max2,* and *baf1max2* were examined. Seedlings were grown on ½ LS medium with different concentrations of BRZ (0, 100, 250, 500 and 1000 nM) for 7 days in dark (A). Hypocotyls were measured using ImageJ (B). Data represent mean ± SEM of 18 seedlings from three biological replicates (n = 18). (C) and (D) BRZ sensitivity assay in the weak light. WT, *baf1, SINAT RNAi, baf1SINAT RNAi, max2,* and *baf1max2* were examined. Seedlings were grown on ½ LS medium with or without 250 nM BRZ for 10 days in weak light (C). Hypocotyls were measured using a ruler (D). Data represent mean ± SEM of 20 seedlings from five biological replicates (n = 20). Only significant differences between genotypes within one BRZ treatment were compared, which were indicated by different letters according to one-way ANOVA Tukey's multiple range tests ($p < 0.05$). Scale bar represents 10 mm. (E) *In vivo* ubiquitination of BES1 from WT, *baf1*, *SINAT RNAi* and *baf1 SINAT RNAi* seedlings. BES1 protein was immunoprecipitated from 7-day-old seedlings by BES1 antibody. The immunoprecipitation product was analyzed by immunoblotting with anti-BES1 and anti-UBQ (rabbit) antibodies.

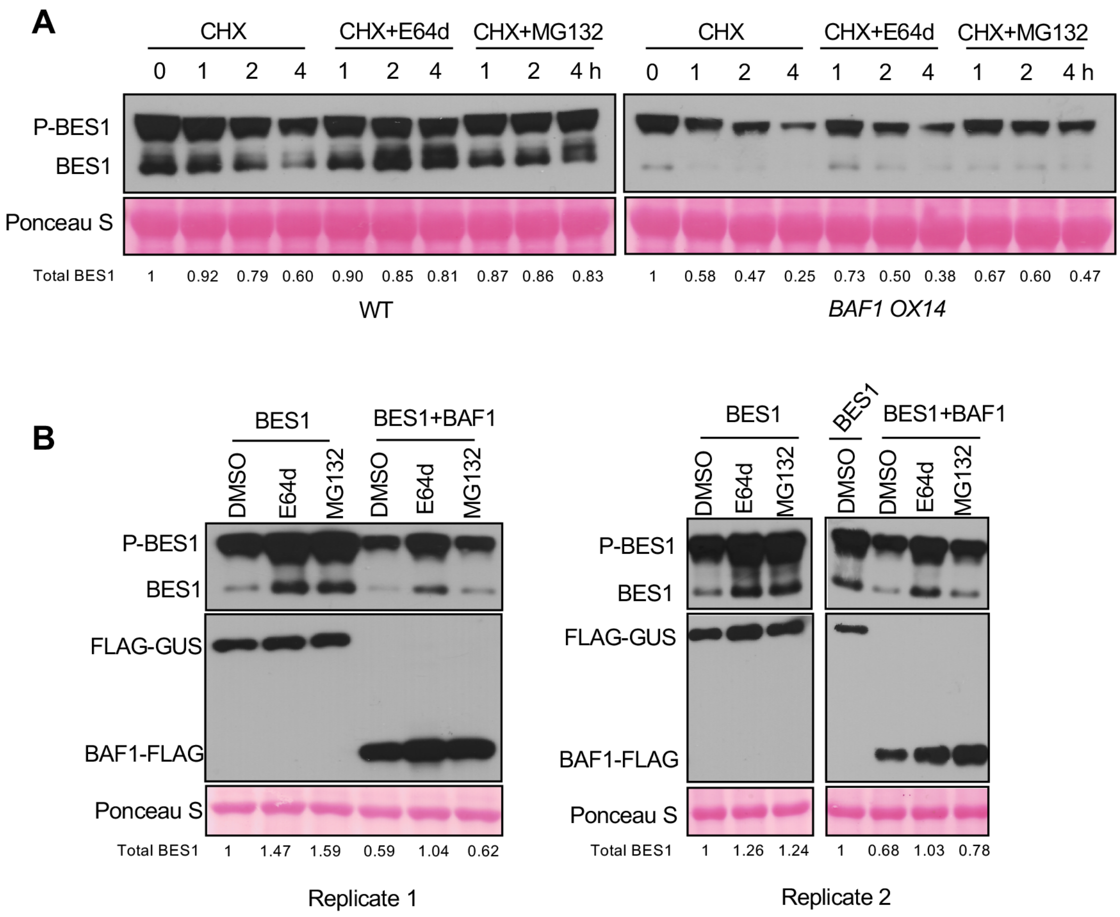

Supplemental Figure S7. The response of BAF1-mediated BES1 degradation to inhibitors in Arabidopsis seedlings and *N. benthamiana*. (Supports Figure 7). (A) BES1 protein stability was examined by treating Arabidopsis seedlings grown on ½ LS media with 1 mM CHX with or without MG132 or E64d for indicated times. WT and *BAF1-FLAG OX14* were examined. Samples were analyzed by immunoblotting with anti-BES1 antibody. (B) BES1 was co-infiltrated with BAF1-FLAG or control vector into *N. benthamiana*. At 12 hours post-infiltration, DMSO, 50 μM MG132, or 40 μM E64d were infiltrated into the same leaf area as infiltrated before. Samples were collected 24 hours after addition of inhibitors and analyzed by immunoblotting with anti-BES1 and anti-FLAG (rabbit) antibodies.
Ponceau S staining serves as protein loading control. Quantified relative band intensity of total BES1 protein was listed below using Image J.

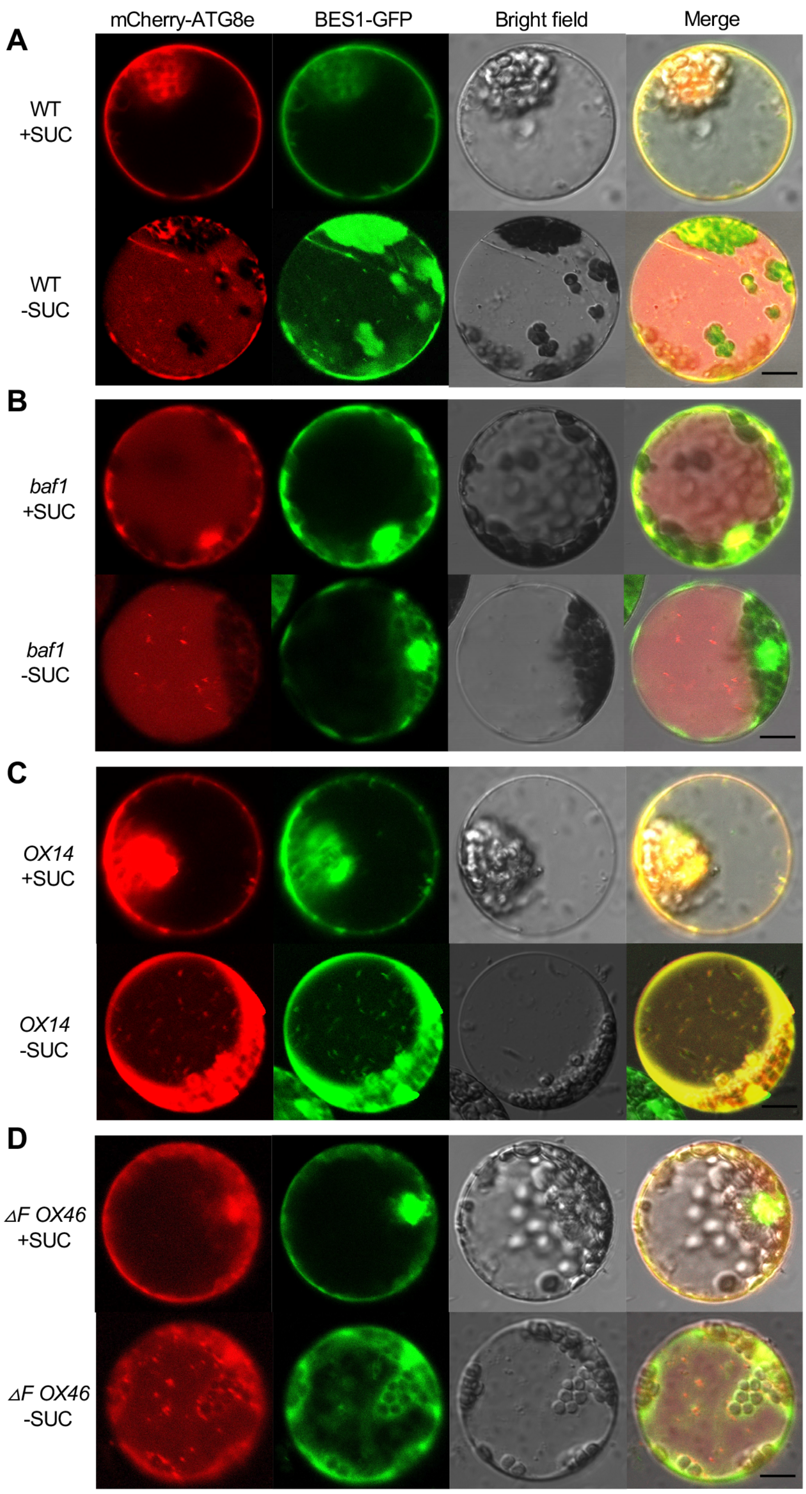

Supplemental Figure S8. The co-localization of BES1-GFP and mCherry-ATG8e in protoplasts from different genotypes. (Supports Figure 7). BES1-GFP and mCherry-ATG8e were co-transformed into WT (A), *baf1* (B), *BAF1 OX14* (C) and *BAF1-ΔF OX46* (D) protoplasts. Protoplasts were treated without or with 0.5% (w/v) sucrose for 36 h before confocal microscopy. GFP and mCherry fluorescent signals were excited with 488 nm and 555 nm, respectively. The signals were then collected using the emission filters of 555 nm for GFP and 640 nm for mCherry. Scale bar represents 20 μm.

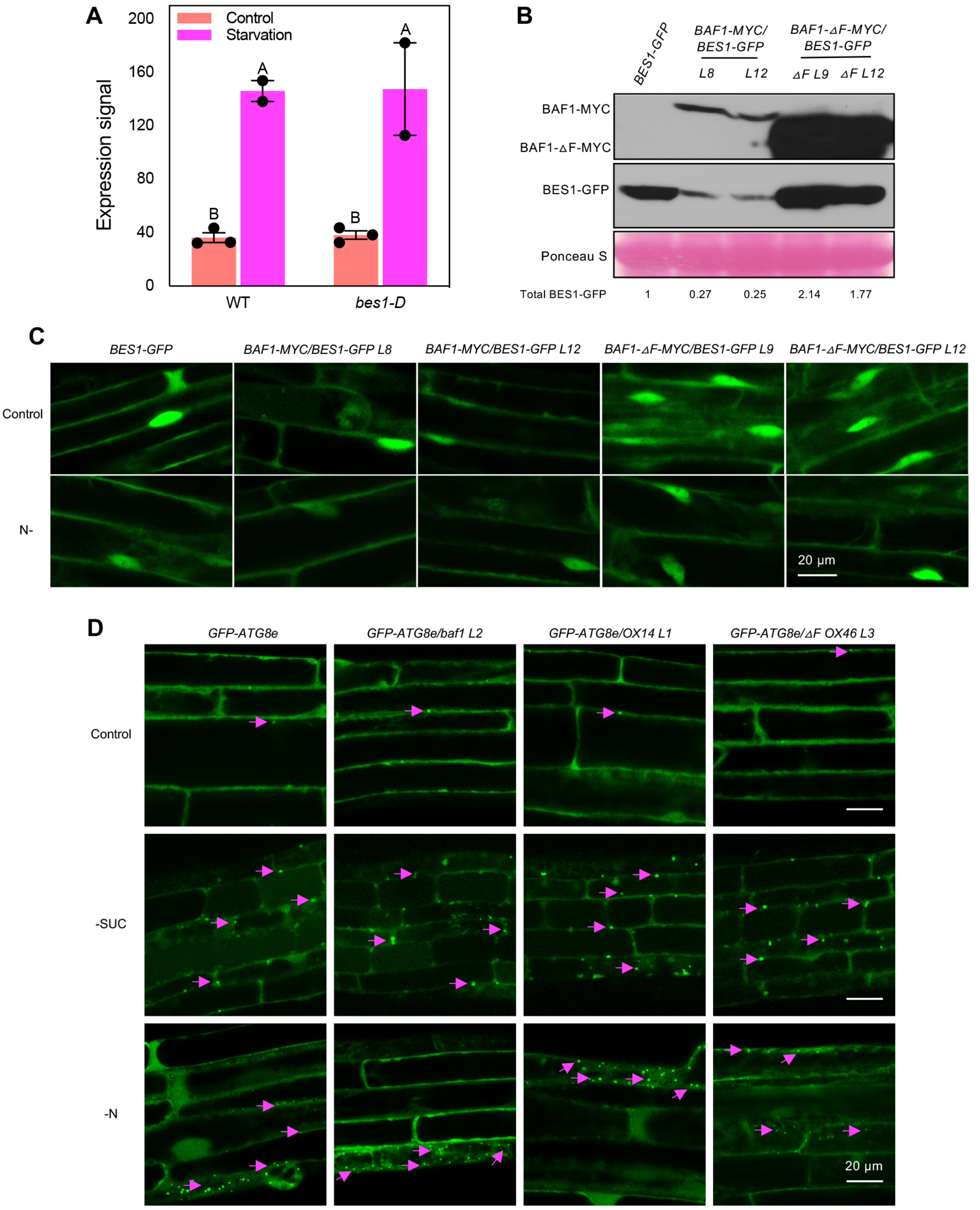

Supplemental Figure S9. BAF1 mediates BES1 degradation largely through autophagy. (Supports Figure 7). (A) *BAF1* gene expression in 4-week-old plants after 5 d dark treatment to cause fixed-carbon starvation. Data were extracted from the published RNA-sequencing dataset GSE93420. Data represents the mean of 3 biological replicates for the control and 2 biological replicates for fixed-carbon starvation. (B) Protein expression in *BAF1-MYC OX* lines and *BAF1-ΔF-MYC OX* lines in *35S:BES1-GFP* background. 7-d-old seedlings were collected and analyzed by immunoblotting with anti-MYC (rabbit) and anti-GFP (rabbit) antibodies. Ponceau S serves as loading control. Quantified relative band intensity of total BES1-GFP protein was listed below using Image J. (C) Confocal images of BES1-GFP labelled puncta under nitrogen starvation for 16 h in the Arabidopsis roots. Scale bar represents 20 µm. (D) Confocal images of GFP-ATG8e labelled puncta in representative lines (from Figures 7H-7I) under sucrose or nitrogen starvation for 3 d in the Arabidopsis roots. GFP fluorescent signals were collected with excitation and emission at 488 nm and 555 nm, respectively. Red arrows indicate the puncta. Scale bar represents 20 µm.

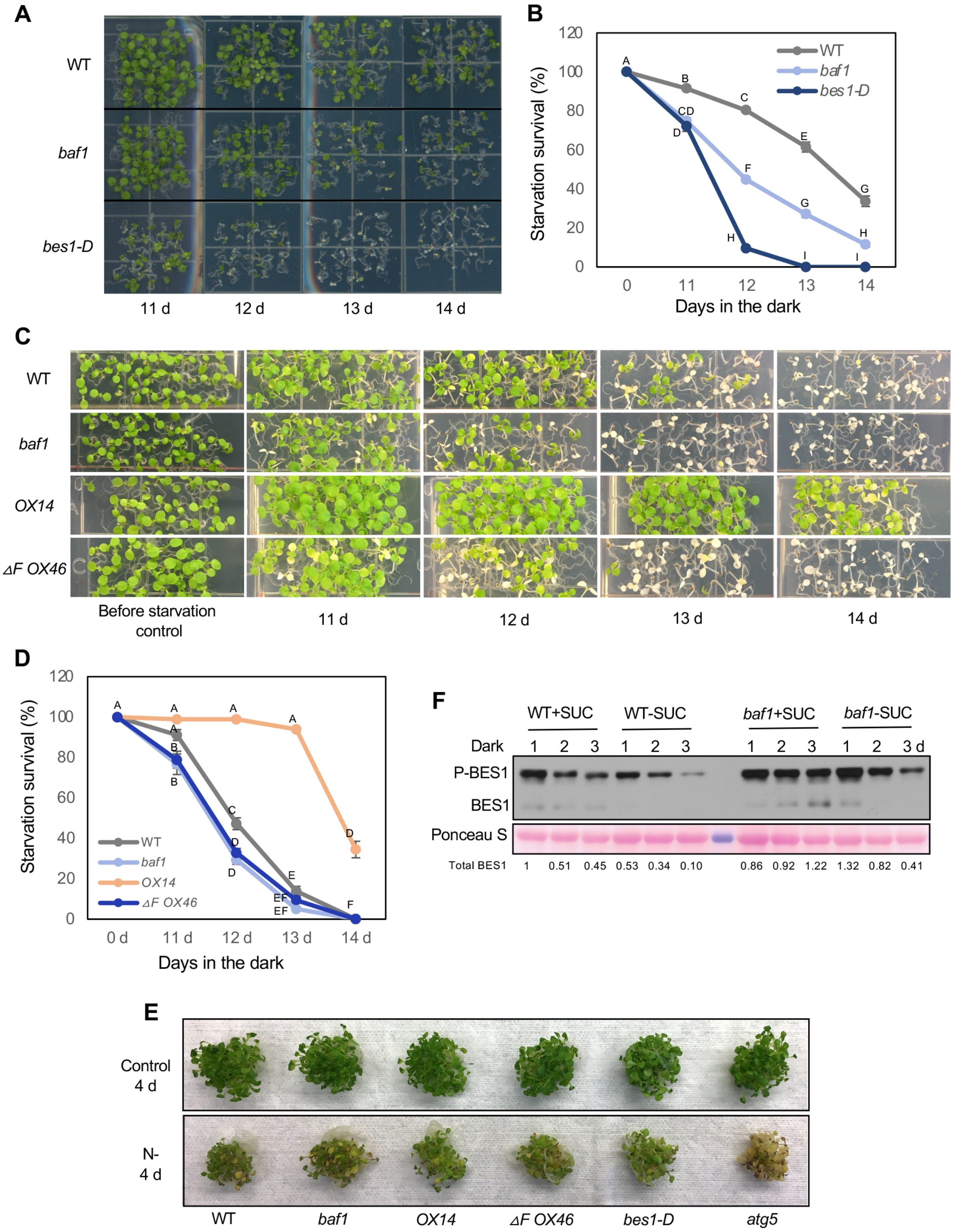

Supplemental Figure S10. BAF1 mediates plant sensitivity to long-term sucrose starvation. (Supports Figure 7 and Figure 9). (A, C) and (B, D) Seedling phenotype and survival percentage after long-term sucrose starvation in the dark (indicated times) followed by 7 d recovery in the light. Seedlings remaining green or with new growth emerging are considered as surviving. Data represent mean ± SEM from 9 biological replicates (n = 9) for (B) and 6 biological replicates (n = 6) for (D); each replicate contains 30 seedlings. Different letters indicate significant difference according to one-way ANOVA Tukey's multiple range tests ($p < 0.05$). (E) Phenotypes in response to nitrogen starvation. Similar numbers of 7-day-old WT, *baf1*, *BAF1 OX14*, *BAF1-ΔF OX46*, *bes1-D* and *atg5* seedlings were transferred to ½ LS liquid with or without nitrogen for 4 days and photos were taken. (F) BES1 protein degradation under sucrose starvation in dark in WT and *baf1* mutant. Samples were analyzed by immunoblotting with anti-BES1 antibody. Ponceau S serves as loading control. Quantified relative band intensity of total BES1 protein was listed below using Image J.

Supplemental Table S1. Primer sequences used in this study.

Primer Name	Sequence
BAF1-F	ATGGAATCGTCTCCGGTAAACTG
BAF1-ΔF-F	ATGCGTCCGCCGTTGAATCTAC
BAF1-R	TCATGGCATCAATGTAAGCTTAGCAT
BAF1-Asp-F	CACCGGTACCATGGAATCGTCTCCGGTA
BAF1-SalI-R	CACCGTCGACTGGCATCAATGTAAGCTT
BAF1-EcoRI-F	CACCGAATTCATGGAATCGTCTCCGGTA
BAF1-BamHI-R	CACCGGATCCTCATGGCATCAATGTAAGC
BAF1-ΔF-Asp-F	CACCGGTACCATGCGTCCGCCGTTGAAT
BAF1-ΔF-EcoRI-F	CACCGAATTCATGCGTCCGCCGTTGAAT
qBAF1-F	GATCGGTGGATTGAAATCGACG
qBAF1-R	GCTTCCCCATCCTTTTAGCAG
qUBQ5-F	ACAATGTGAAGGCGAAGATCC
qUBQ5-R	AGCTTAACCTTCTTATGCTTGTGC
qACTIN2-F	TCAACCAATCGTGTGTGACAAT
qACTIN2-R	CACCATGCTCAATAGGATACTTCAAG
qAT3G55920-F	GCAAACTCGGGACCAGACTC
qAT3G55920-R	GCTTTGGGCACTCCACTGT
qAT3G05170-F	AATCGGAAGGGAATCTCGACAC
qAT3G05170-R	GTAGATCGAGTCCGATCGTACG
qAT3G30720-F	ATGAAGACCAATAGAGAGCAGGAA
qAT3G30720-R	TCAGTAGTTGTAGAACTGAAGCCC
qAT5G15290-F	ATGAAGTCCGGTCAGGCTG
qAT5G15290-R	GCTTGGAACCGTATGAATTGAGT
qAT3G55240-F	ATGGTGCAGCACATGATAGAGA
qAT3G55240-R	CATTTGCTCTTGTTTGCTTTGCC
qAT5G05250-F	AGTTGTGGCTGTGGTTTTGAC
qAT5G05250-R	GAAATGCGTACACCGCATACC
qAT5G01740-F	ATGGAAGCACACAACGTAGAGAT
qAT5G01740-R	GTGTTGACAATCGTGCGGT
qAT1G66100-F	TGAATACTGCAAGTTGGGGTG
qAT1G66100-R	CTACGCATTTTCAACTGCATTCA
qAT5G65980-F	ATGGGTTTCTTAGAGTTGTTGGAGG
qAT5G65980-R	CAACATTTATGGGCATAAACCACCAT
qAT1G79640-F	ATGCCATACATGTCTGGTGGT
qAT1G79640-R	GTCTCCCAACTTGACTGCACC
baf1-LP	CAACGAATATCCGACCGTACATC
baf1-RP	ATCTCTAACGGCATTCTACCTGCT
GABI-LB	ATAATAACGCTGCGGACATCTACATTTT
atg5-LP	ATTTGCTATTTGTTTGGCACG
atg5-RP	TACCGTTCATGACAGAGGTCC
LB3	TAGCATCTGAATTTCATAACCAATCTCGATACAC
BES1-F	ATGACGTCTGACGGAGCAAC
BES1-R	TCAACTATGAGCTTTACCATTTCCAAG
BES1-F1-EcoRI-F	CGGAATTCATGACGTCTGACGGAGC
BES1-F1-KpnI-R	GGGGTACCTTAAGGTAGAGGCTTGTGTCCC
BES1-F2-EcoRI-F	CGGAATTCACTTATCGCAAGGGACACAAGCC
BES1-F2-KpnI-R	GGGGTACCTTAAGCCATGGACATGGATTGT
BES1-F3-EcoRI-F	CGGAATTCACCAAACAATCCATGTCCATG
BES1-F3-KpnI-R	GGGGTACCTTACACGAGATTGAAGGTAGGCG
BES1-F4-EcoRI-F	CGGAATTCCCAACCTCGCCTACCTTC
BES1-F4-KpnI-R	GGGGTACCTCAACTATGAGCTTTACCATTTCC

Supplemental Table S2. Information on constructs used in this study.

Recombinant DNA	Source	Identifier
pET42a GST	Novagen	70561
pET42a GST-BAF1	This study	N/A
pET42a GST-BAF1-ΔF	This study	N/A
pETMALc-H MBP	Pryor and Leiting, 1997	N/A
pETMALc-H MBP-BES1	Yin et al., 2002	N/A
pETMALc-H MBP-BES1 F1-F4	This study	N/A
pGBKT7	TAKARA	630443
pGADT7	TAKARA	630442
pGBKT7 BES1	This study	N/A
pGBKT7 BES1-D	This study	N/A
pGADT7 BAF1	This study	N/A
35S:BES1	Yin et al., 2002	N/A
35S:BES1-GFP	Yin et al., 2002	N/A
35S:BES1-D-GFP	Yin et al., 2002	N/A
35S:mCherry-ATG8e	Bassham Lab	N/A
35S:GFP-ATG8e	Bassham Lab	N/A
35S:BAF1-FLAG	This study	N/A
35S:BAF1-ΔF-FLAG	This study	N/A
35S:BAF1-MYC	This study	N/A
35S:BAF1-ΔF-MYC	This study	N/A
35S:BAF1-GFP	This study	N/A
pGWB412 FLAG	Walley Lab	N/A
pGWB406 GFP	Walley Lab	N/A
pGWB412 FLAG-GUS	This study	N/A
pGWB406 GFP-GUS	This study	N/A
pGWB406 GFP-BZR1	This study	N/A
35S:MYC2-FLAG	Guo et al., 2018	N/A
35S:TUBE-FLAG	This study	N/A
35S:cYFP	Yu et al., 2008	N/A
35S:nYFP	Yu et al., 2008	N/A
35S:BES1-nYFP	Wang et al., 2014	N/A
35S:MYC2-nYFP	Guo et al., 2018	N/A
35S:BAF1-cYFP	This study	N/A
35S:BAF1-ΔF-cYFP	This study	N/A

Supplemental Table S3. Information on antibodies used in this study.

Antibodies	Source	Identifier
Rabbit polyclonal anti-BES1	(Yu et al., 2011)	N/A
Rabbit polyclonal anti-GFP	Lab made	N/A
Rabbit polyclonal anti-GFP	Invitrogen	A11122
Rabbit polyclonal anti-FLAG	Sigma-Aldrich	F7425
Rabbit polyclonal anti-c-MYC	Sigma-Aldrich	C3956
Rabbit polyclonal anti-IWS1	Lab made	N/A
Chicken polyclonal anti-Ubiquitin	(Pratelli et al., 2012)	N/A
Mouse monoclonal anti-FLAG	Sigma-Aldrich	F1804
Rabbit anti-K48-linkage polyubiquitin	Cell Signaling Technology	4289S
Rabbit anti-K63-linkage polyubiquitin	Cell Signaling Technology	5621S
Rabbit polyclonal anti-Ubiquitin	Cell Signaling Technology	3933S
Mouse monoclonal anti-MBP	New England Biolabs	E8032S
